# Stability of Resistance of Maize to Ear Rots (*Fusarium graminearum*, *F. verticillioides* and *Aspergillus flavus*) and Their Resistance to Toxin Contamination and Conclusions for Variety Registration

**DOI:** 10.3390/toxins16090390

**Published:** 2024-09-10

**Authors:** Akos Mesterhazy, Balazs Szabo, Denes Szieberth, Szabolcs Tóth, Zoltan Nagy, Tamas Meszlenyi, Beata Herczig, Attila Berenyi, Beata Tóth

**Affiliations:** 1 Cereal Research Non-Profit Ltd., P.O. Box 391, 6701 Szeged, Hungarytamas.meszlenyi@gabonakutato.hu (T.M.); atti.berenyi.91@gmail.com (A.B.);; 2 Hungarian Maize Club, Kazinczy Street 12, 8152 Kőszárhegy, Hungary; magyarkukoricaklub@me.com; 3 Bonafarm Dalmand Inc., Felszabadulás Street 42, 7214 Dalmand, Hungary; 4 Bonafarm-Babolna Feed Ltd., Laboratory Branch, 2942 Nagyigmand, Hungary; Bea.Herczig@btakarmany.bonafarm.hu

**Keywords:** maize, *Gibberella* ear rot, *Fusarium* ear rot, *Aspergillus* ear rot, stability, resistance, resistance to toxin accumulation

## Abstract

All major ear rots (*F. graminearum*, *F. verticillioides*, and *Aspergillus flavus*) and their toxins are present in maize of preharvest origin in Hungary. Resistance can be an important tool in reducing the infection and toxin contamination from these rots in maize. Previous results identified resistance differences in maize hybrids that were suitable for use in evaluating their risk from toxigenic fungi and their toxins. During the tests, two methodical improvements were achieved: the use of three isolates of the fungus secured and a more precise estimation of resistance to ear rots and their resistance to toxin accumulation or overproduction. The improvement in sampling and the tests of subsamples made the evaluation for the statistics much more exact. This way, we were able to reduce the Within value, providing a statistically more reliable method of evaluation. Earlier data had confirmed that toxin contamination could not be predicted well from visual ear rot severity data. Contradictory results for hybrid ranking were often identified between isolates. The resistance to disease and toxin contamination is not generally valid. The new suggested methodology compares the performance of hybrids in a large number of epidemic situations to identify adaptable hybrids that can respond to diverse conditions; therefore, the stability of resistance and toxin response is decisive information to evaluate risk analyses. The increased number of disease toxin data allowed for lower LSD 5% values for toxins, a much finer analysis of toxin overproduction and underproduction, and a wider database for stability analyses. This way, we obtained important additional separated information about resistance to accumulation of toxins and about maize resistance to these pathogens that is suitable to provide much more reliable testing than was possible until now. Globally, about 50–100 million metric tons can be saved by excluding susceptible hybrids from commercial production.

## 1. Introduction

The maize is most exposed to toxigenic fungi compared to other cereals. The ear rots GER (*Gibberella* ear rot), FER (*Fusarium* ear rot), and AER (*Aspergillus* ear rot) that affect maize in Hungary have been recognized for decades [1,2,3]. Regarding GER and FER, the origin of these toxins has never been explored in the international and national literature, but aflatoxin has been investigated. Christensen and Kaufmann [4] classified it as a storage pathogen without preharvest significance based on the low-rate occurrence of *A. flavus* in field samples. However, US research clarified that preharvest aflatoxin is present in the Southeastern US [5,6]. For now, it is widely accepted that aflatoxin is widely produced preharvest in dry and warm areas [7]. The aflatoxins in maize occurred in Hungary in stored maize, and *Aspergillus flavus* isolates from maize were verified to produce aflatoxin [8,9]. Following the aflatoxin epidemic in 2012, which mostly affected the dairy industry, the idea was raised that preharvest contamination could have contributed; the first publication reporting preharvest aflatoxin occurrence was published in 2022 [10]. Another paper [11] analyzed 17,000 toxin data from maize for DON, FUMB1, and AFB1 between 2012 and 2017 and showed that the southern and eastern parts of Hungary showed more contamination from all toxins than the other parts of the country. As Hungary is located at the crossroads of the Atlantic, Mediterranean, and Continental climate zones, all three fungal species cause epidemics with significantly higher contamination than the EU limits are. Therefore, plant resistance to all of them should be monitored.

In the analytic analyses, the multitoxin levels reported a sharp increase [12,13,14,15]. The global maize yield is estimated for 2022/2023 to be 1165 million metric tons [16] and is estimated to be 1223 million tons for 2023/2024. The FAO estimated the damage from toxigenic fungi affected 25% of the harvested yield [17]. Eskola et al. [18] verified this number to be 25%. Mesterhazy et al. [19] estimated from preharvest and postharvest at least 20%, and this shows that the control of both types of losses is equally important; the total, physical, and quality damaged loss is about 300–350 million tons and this crop is the most susceptible to toxigenic fungi in cereals [20].

The outbreak of these epidemics has three basic determinants: susceptibility in a variety, a high concentration of the causing agents in the environment, and an environment favoring epidemic development. This is the so-called disease triangle. When all three components are present, the epidemic breaks out. The clear conclusion is that we need more resistant genotypes. Toxin control during harvest is useful because, based on rapid methods, the grains can be separated for toxin contamination and stored separately. The grower has feedback on whether the hybrid used was the right one or not. These data also provide feedback for the breeder. EU regulations [21,22,23,24,25] relate to natural (spontaneous) infection. Artificial inoculation has the advantage that the data are comparable, and the ranking is much more reliable [26,27].

Therefore, the variety registration process is vital, allowing susceptible and highly susceptible hybrids to be banned from commercial production. The tests that we have performed over the last 15 years clearly show that about 80% of hybrids should not be under commercial production [28,29,30]. In all breeding companies, low and high-risk hybrids were identified with a very wide variability. We have the impression that even the firms have moderate knowledge of their own hybrids. The DEKALB hybrids [31,32] are marketed with resistance data to four diseases, but no toxigenic fungi are mentioned. The Pioneer hybrids list in South Africa [32] presents resistance data to GER, FER, and Diplodia ear rot, but food safety risk data for possible toxin exposure are not mentioned. The Corteva Agriscience catalog (2019) [33,34] in Hungarian mentions several hybrids of good ear health, but in the summary tables, no toxigenic fungi are mentioned. Other firms [35,36] mention disease resistance but not specifically resistance to ear rots, and the word “toxin” is not mentioned at all. As the methodology is often not reliable [37], this speaks for a more developed methodology. As the Earth’s resources are not endless, the rate of healthy grain should be significantly higher than today. For this reason, the gap between variety breeding, agronomy practice, and scientific research should be bridged.

Most research on ear rot in maize has used one single isolate or a mixture of isolates but, in both cases, one inoculum. This suggests that it is generally believed that this is enough for phenotyping maize genotypes in terms of resistance and toxin production [13,37]. As the experimental results do not support this view [28,29,30], we had many hybrids with contrasting responses to the two isolates used; this led us to increase the number of isolates used to three. Miedaner et al. [38] published a paper about resistance to 8-8 isolates of *F. graminearum* and *F. verticillioides* in two susceptible and three resistant inbreds. They concluded that the different inbreds showed very similar results in response to the isolates of the two species; therefore, one isolate should be enough to test the resistance of maize to these two species. They also performed toxin analyses, and the amount of toxin contamination was high; therefore, it became possible to differentiate the responses among varieties.

In earlier papers, we checked the resistance of maize to fifteen isolates of *F. graminearum* and *F. verticillioides*; the somewhat differing responses led us to modify how we ranked the hybrids tested [13,39]. The use of a single isolate or mixtures of isolates was not an accident in the international literature; similar conclusions have also been drawn in wheat [40] and maize [27]. The reason is that when there are no specialized races in these pathogens, any isolates have the same value. On the other side, all authors found large variability in the aggressiveness of *Fusarium* spp. [41,42] The aggressiveness level varies between years but may change between inocula of the same isolate [43]. However, it is an experimental fact that experimental results do not verify this idea [28,29,30]. Therefore, varying aggressiveness and inconsequent hybrid reactions cause problems in determining resistance in terms of not only resistance ranks but also resistance levels. Looking at the data of Miedaner et al. [38], it is clear that aggressiveness significantly influences the disease severity and also, in some cases, the disease rank. The same genotype can be considered resistant or susceptible depending on the isolate, as shown in their Figure 1. Is this important? It seems so because genetic research requires exact phenotyping data as far as possible; otherwise, QTLs can be incorrectly determined as false-positive without real genetic background or negative. In a breeding program, this causes problems. Identifying false-positive or -negative results is very probable. This is another reason why exact and reliable data are needed; we need more data to support more solid conclusions.

The polygenic nature of the resistance is well supported [37,41,44,45]. Most of the QTLs are weak and often not validated, but several QTLs with larger effects have also been identified. Data show significant isolate, environment, and genotype interactions; therefore, the stability of the response has emerged as being of key significance [28,30,46]. For this reason, we need to provide a larger dataset for stability analysis in genotypes, inbreds, or hybrids, and experimental data support this conclusion. [28,29,30,38]. This was one of the reasons why we increased the number of isolates to three in this paper. For polygenic traits, such as yielding ability, tests are conducted in many locations, and based on the results, we can determine whether a variety adapts well. Low variance means higher stability and higher adaptation ability that a variety ranks within the first 20–25% of the hybrid set. A high variance means that performances range from very good to very bad (like percentages between 5 and 85). The performance of badly adapted hybrids cannot be forecasted. To decide this, we need to have a reasonably high amount of data in order to form an opinion about the stability or instability of the hybrid.

There are several previous reports about possible common resistance to different toxigenic species. The resistance to *F. graminearum* and to *F. culmorum* showed consequently a close relation, and this is supported by our results [28,29,46,47]. Resistance to *F. graminearum* and *F. verticillioides* or *F. verticillioides* and *A. flavus* [13,37,45] identified agreements in individual hybrids, but no general agreement was found (r = 0.5–0.6). As they were one inoculum test, the reliability can be problematic. We concluded that a generally valid combined resistance to the three pathogens (*F. graminearum*, *F. verticillioides*, and *A. flavus*) does not exist, but about 10–15% of the hybrids have good resistance to all three main species, including their toxins, 0–10% have high or very high susceptibility to all three pathogens, and the rest vary greatly. It is important that the resistance differences between hybrids are highly significant, often tenfold or higher; this makes it possible to screen hybrids to the three pathogens and suggest only the plus variants for commercial production.

The objective of this paper is to develop a methodology for resistance screening and breeding programs with more developed phenotyping, with the following components: (1) testing three parallel isolates of three toxigenic species for resistance to disease and resistance to toxin contamination; (2) testing the stability of the resistance responses; and (3) improving toxin analysis to reduce the Within value in ANOVA and render the LSD 5% determination more useful in analyses the resistance and toxin contamination. (4) Suggest a risk analysis that provides reliable information for toxin risks.

## 2. Results

### 2.1. Ear Rot Data Visual Scores

The artificial inoculation data for the maize hybrids (Table 1) show large differences between pathogen species, but the mean aggressiveness levels are close for the three isolates of the same species. The highest stability (lowest variance) against all toxigenic species and their isolates was found in Cadixxio Duo. The P9415 showed only a slightly worse performance. In spite of the close genotype means, Fg3 presented a threefold higher number than Fg4, and the opposite situation was observed for SY Zephyr, which produced double the infection severity of Fg3. Fornad, however, presented the same numbers. Such inconsequent behavior was also found for *Aspergillus* in Cadixxio Duo and Korimbos. This means that one set of data for one isolate for two years may not fit with the genetic background, and false positive and negative results may occur, indicating problems in evaluating resistance level for resistance or QTL mapping tests in which different QTLs might be identified for different isolates. The correlations (Table 1) show significant relations between responses in Fg3/Fg4 and Fg4/Fg6, but the correlation for Fg3/Fg6 is not significant. The situation is similar for *F. verticillioides*. Here, Fv1/Fv5 was not significant. The responses to the other two isolates show more similar data, and these correlations are significant. For the least aggressive, *A. flavus*, no significant correlation between responses to different isolates was found. We think, therefore, that a higher number of isolates leads to more reliable results for resistance levels than for any isolate alone. Regarding the correlations between isolates of the three toxigenic species, out of the 36 correlations, only 4 were significant: Fg6/Fv5 and Fg6/Fv8. No significant correlations were found between the control ear rot and artificial inoculation data.

The ANOVA table (Table 1) shows significant main effects, except for isolates. Their sums were nearly the same, so a significant influence was not probable. The genotype differences across the three species and control were significant., so the artificial inoculation significantly increased the disease severity compared to the natural infection. The two years showed different disease severities. *F. graminearum* had a much higher aggressiveness than *F. verticillioides*, and *A. flavus* was significantly less aggressive than *F. verticillioides*. This confirmed the earlier findings. Of the two-way interactions, those including the isolating effect were not significant, but the others showed different responses in different years. The three toxigenic species reacted differently between the two years (AxB), and their infection severity also differed between the two years (BxD). At the same time, the hybrid ranking was also different for the different toxigenic species (AxD). Also, the ANOVA supported the significant interaction between fungal species, so we had to analyze the data separately according to toxigenic species.

The *F. graminearum* data (Table 2) show fourfold significant genotype differences. According to the means, the differences between genotypes are more than fourfold. The variance shows the stability of performance. Cadixxio Duo shows very low infection severities in all situations, and it has the lowest variance, i.e., the highest stability. The second P9415 also results in low numbers for infection severity; its stability is slightly weaker. We observed high stability for Fornad at a significantly higher susceptibility level, indicating a stable higher-than-medium infection severity every year. Korimbos was used as the resistant control, as was the case in the last nine years, and this year, it had a medium performance. In two cases, however, it behaved as highly susceptible. Therefore, its adaptation ability is problematic. In terms of adaptation, P0725 was the weakest; thus, it has the highest variance and highest instability in response. The susceptible control DK4541 was the most susceptible, but it was more stable than the less susceptible, and it was badly adapting P0725. The maximum and minimum stability differed 15fold. The variety differences are highly significant (Table 2), but the column means they do not show significant differences, and the interaction between genotypes and year/isolate effect is not significant again, showing rather good stability in responses. The inconsequent correlations (Table 2) are due to the mixture of unstable and stable performance (adaptation) hybrid responses to the isolates themselves. The correlations are seldom significant (3 out of 15). In three cases out of six, the individual isolates correlate significantly with the mean. With a larger database, the rate of significant correlations would probably increase as the limit for correlation decreased. This is one reason why we should have as large a database as possible.

The *F. verticillioides* ear rot data (Table 3) also show significant genotype differences. Sy Zephyr had the lowest infection severity and the highest stability. Five of our other hybrids did not differ significantly from Sy Zephyr. The infection severity was significantly lower in 2021 than in 2022. The differences between isolates were also significant. The variety ranks were not the same; in all cases, we observed variations, from no difference to double difference. Four hybrids had a higher infection rate, and the instability of responses was high. Among the medium- or high-infected hybrids, no one had good stability; this differed from the finding found in *F. graminearum*. The difference between the stability of the most stable hybrid and the least stable hybrid was 50-fold. The year/severity interaction might be different for the different pathogens (Table 3). The correlations between the responses to the six epidemic situations were mostly not significant (Table 3). In 2021, only the F5/F8 correlation was significant and non-correlated with the means. In 2022, the correlations were closer, and the relations with the mean became highly significant for the same isolates.

*Aspergillus flavus* shows a similar pattern (Table 4); 2021 was not an *Aspergillus* year, but 2022 had the highest numbers compared to the data for the preceding years [10]. The highest mean for the most susceptible hybrid was close to one percent of ear coverage. The lowest was 0.2, meaning that a fivefold difference was recognized with significance at *p* = 0.05. Four genotypes had a variance lower than half the experimental mean (bold print), accompanied by a low infection severity across all epidemic situations. For stability (variance), the difference between the maximum and minimum hybrid values was 26-fold. As the isolates for the two years were the same, the highly different infection levels cannot be explained by genetic differences between the isolates of pathogens used. This is due to the ecological sensitivity of *A. flavus* disease development. In spite of this, the hybrid differences were significant, but the genotype/isolate x year interaction was significant, indicating different rankings in the years and isolates (Table 4). When we examine the correlations between the six AER datasets, in most cases, the data for the same isolates for the two years do not correlate (Table 4). However, in three cases, the data correlate significantly with the means of the six columns.

The general means for the three toxigenic species, together with the controls (Table 5), show the ranking and resistance risk classification of the hybrids. The hybrids are ranked according to the mean reaction. As resistance is mostly not connected, and the correlations also show this clearly (Table 5), independent ANOVAs were conducted for each toxigenic fungus. This type of general analysis is not without value, as it helps identify hybrids with low infection values and good resistance to all toxigenic fungi. This is shown by the LSD 5% value for the means; the individual LSD 5% values, are at the column means. This analysis was also necessary because the mean pathogenicity of the toxigenic fungi largely differed. Therefore, the ranking will be determined by the most aggressive species (*F. graminearum*).

We have three hybrids that fall into the low and low-to-medium classes, shown with dark- and light-green highlighting and their names printed in bold. Fornad raises a question: for *F. graminearum*, the infection severity is above average, but for the others, it is in the best category. The resistant control is Korimbos, based on its behavior over the past six years, was good for *F. graminearum* and *F. verticillioides*, much less for *A. flavus*, and its high natural infection is also a risk factor. The correlations among levels of resistance to the different toxigenic fungi are not significant. The main reason for this is due to the different resistance backgrounds in most hybrids. The resistance to different fungal species is mostly not connected, and the correlations show this clearly (Table 5). Therefore, independent ANOVAs were conducted for each toxigenic fungus. The LSD 5% values are shown in each column. The most aggressive species, *F. graminearum*, determines the means; therefore, the less aggressive toxigenic cannot provide significant differences, even if we have them.

We have three hybrids that are in the low and low to medium class with dark and light green highlighting; their names are printed in bold. Fornad is a question. For *F. graminearum*, its infection severity is above average. However, this is for the others in the best category. Here, the toxin contamination will be decided. The resistant control Korimbos behaved mostly, so in the past six years, it has had good resistance to the two *Fusarium* spp. But not to *Aspergillus* ear rot. The not-treated control had higher infection than we would prefer. P9978 is an exception. It has only yellow ranking across data, and the last three hybrids show a mixed ranking for the different toxigenic fungi.

The natural infection severities (which appeared to be independent of the directly toothpick-infected area) of *Fusarium* and *Aspergillus* showed very similar results to the control. The column means for the control were between 0.26 and 0.29 ear rot %. For the artificially inoculated versions of the three toxigenic species, the means varied between 0.18 and 0.40 (Table 6); only one column showed a significant difference from the control mean (0.275) for Asp. 3. Fg3 differed significantly from Fg4. There were four hybrids with low infection severity and low variance, which are valuable traits for the future (bold printed), and P9405 is probably connected to this group. The variance is generally very similar, except for P9978. This means that in these years, the background infection was very similar to that in the control. So, the independent infection beyond the toothpick-inoculated area was not significant. All species showed significant variety differences. ANOVA was performed for all toxigenic species separately. Here, only the LSD 5% data are given. This means that the column data are in the same range without significant differences. Among the correlations (Table 6), a larger number are significant. Out of 55 cases, 18 correlations are highly significant. This also shows how unreliable it is to determine resistance based on the natural infection (Table 7).

The natural ear rot infection, according to toxigenic spp. shows sixfold significant differences (Table 7). The stability ranges between 0 and 0.02 for the hybrids, showing rather uniform performances, much better than the individual isolates, as shown in Table 6. The additional natural infection of the inoculated ears was also on the same level as the artificially inoculated ears, not only at the isolate level but also at the species level. The variance, except for Korimbos, was very high, except for one, where the natural infection showed good agreement. The correlations between the *Fusarium* spp. inoculated, and the control was very high, but the natural infection in the *Aspergillus*-inoculated ears presented no significant correlation with any other toxigenic species. (Table 7). This means that, in this case, the artificially inoculated ears did not amass more infection than the naturally infected control ears. This means that no antagonistic responses between species and their isolates could be detected. Of course, this does not mean that the toxin data would behave the same way.

The natural *Aspergillus* ear rots on the check, and the artificially inoculated ears (Table 8) present significant genotype differences with threefold differences. The control had 0.13% severity, the *F. graminearum* and *F. verticillioides* ears somewhat more (without significance). However, the *Aspergillus* infection on the *Aspergillus* artificially infected ears was three-fold larger than we had in the control. The reason is not known, but the seemingly independent infections might not be fully independent of the artificial inoculation as was found in *Fusarium*. This will require further research to find an answer. Having the toxin data, we might have more support for a possible explanation.

### 2.2. Toxin Contamination

The presentation of mean toxin data across years (Table 9) shows the artificial and natural contamination data together. As the weather conditions were different, Table 15 also shows the yearly data in detail.

When we compare the hybrid performance of the three isolates in response to the same toxin (Table 9, for *F. graminearum*, five hybrids gave similar responses to the three isolates, five contradicted. In *F. verticillioides*, 4 hybrids were stable; six gave different values. For *Aspergillus*, two were stable, and 8 not. The phenomenon is the same as that found for ear rots; the hybrids often provide contradictory results to the different isolates of the same toxigenic species we have found for the ear rot data. The hybrids showed fourfold differences across all data; P9415 showed the lowest mean and the lowest variance, e.g., the highest stability. Except for one fumonisin data, the others were good or acceptable. DKC 4541 and Cadixxio Duo had mostly good data; in many cases, the diversity within species was considerable. For the other hybrids, especially the aflatoxin data, it caused mostly anxiety, but in several hybrids also, DON and fumonisins were considerable. In mean performance, the best hybrids gave below 2.0, the worst 6.7 with a variance between 2.7 and 68, more than a twentyfold difference; the year 2022 was an aflatoxin year. The year effect for all toxins will be separately shown in Table 15.

ANOVA (Table 9) showed that all effects (main and their interactions) were significant at *p* = 0.001 or higher when tested against the Within. As the four-way interaction was significant, the F values were also tested to the A x B x C x D interaction. The hybrid differences, years, isolates, and toxins differed significantly. However, at this level, the interactions containing hybrid effects (A x B, A x C, and A x D) were not significant between the variety and other traits, even if their F value compared to the Within value was significant. The interactions between years, isolates, and toxigenic species were significant, showing that the data strongly differed in their combinations, but the hybrid effect was relatively stable. As the correlations were generally not significant among toxin levels between isolates and toxigenic species, for all toxins, a different analysis was performed to determine the LSD values for the individual toxins. Of the three-way interactions, only the B x C x D interaction was significant; the other interactions did not significantly differ from the four-way interaction.

From the correlations (Table 9), we see that of the 66 correlations counted, only five were significant. There is no generally valid correlation matrix that would be suitable to help forecast toxin contamination, even based on one result set to an isolate for the other.

For the stability test, we included data from the two years, both from artificial and natural origins; for the risk analysis, both are important. These tests were made for all three toxins separately. The DON data show very large differences; 0.11 mg/kg is the minimum, and the most susceptible hybrid had 12.41 mg/kg (Table 10) with an LSD 5% value of 0.20 mg/kg. Two hybrids have a lower mean than 1, and one just above it (1.13 mg/kg). For them, the ear rot is low, stability is good, and the variance is lower than 10, but the highest values are close to 3000 or higher. The correlations (Table 10) are mostly not significant, indicating that if any of the isolates is selected, its forecasting power for response to the other isolates and other ecological situations is problematic.

The fumonisin contamination (Table 11) in the artificial inoculation results shows rather good similarity between the two years; the mean data show sixfold differences between hybrids with an LSD 5% 0.11 (Table 11). The hybrid differences were significant across the eight epidemic situations, and the stability of the data varied between 1.8 and 137. It could be proven that there are hybrids with stable low infection severity and low variance, e.g., high stability, besides the badly adapted hybrids without valid forecast chance. We prefer the first group. The correlations except four of 28 are not significant; this is in agreement with earlier findings.

The aflatoxin data for the two years show the behavior of the varieties in eight epidemic situations (Table 12). The years 2021 and 2022 differ significantly. Here, the three isolates presented significant differences, but Isolate 3 and the controls were close to zero or zero. The other two isolates caused variable amounts of AFB1 contamination; only one hybrid was free of contamination (lower than the detection limit or zero). The year 2022 was very different; the AFB1 contamination was very high, with significant differences between isolates. For 2020, we see higher numbers [30], but this year was the first where all the hybrids showed a naturally higher contamination than the EU limit value. It is clear that, based on one isolate, no correct resistance estimate can be made. However, when we see the variance in the responses in the hybrids, we can identify about four hybrids that have low variance and a low AFB1 level. DKC5542 is clear; except for one data, all are in the lowest quarter and have low-to-medium exposition. P9415 has one critical data; the rest are fine. Cadixxio Duo does not have an orange ranking; it also has a low mean, and the natural contamination value is the second best. The correlations (Table 12) show that in 2021, Af1 and Af3 correlate with r = 0.91, but by plotting the data, the same correlation was not significant the next year. As Af3 caused no contamination, no correlation could be counted. Only two of the 28 correlations are significant, so it is not possible to predict future behavior based on the correlations for the hybrids calculated based on any single isolate or dataset. However, it was possible to identify hybrids with low mean performance and low variance; even 1–2 higher data might occur.

We mentioned that in earlier tests, the toxin contamination for a percentage of visible infection showed large differences, and this is also the case in this test (Table 13). In DON, the difference between the maximum and minimum rates was 28-fold, for FUM 18-fold, and for AFB1 15.23. This high variability is likely one source of the low correlations.

It does not seem to be resistance-related. One of the most resistant Cadixxio Duo variants produces a fivefold higher DON value for a % of infection than the second most resistant hybrid, P9415, which is a better DON producer. As the phenomenon significantly influences the toxin contaminations and lowers correlations, its understanding needs more research work. We should mention that there are two ZD (zero divider) designations instead of numbers. In these two cases, no visual infection was detected; therefore, this number is zero; hence, the ZD designation, as dividing by zero, is not possible.

### 2.3. Influence of Drought

We should mention that the drought in 2022 caused severe yield losses. The draught response was measured by the number of harvested ears from 30 plants in a row in ten replicated trials. The second index comprised the number of ears of close-to-normal size, evaluated on a scale from 1 to 5; this latter index showed the best variant. The correlations between drought indexes and artificial fumonisin and AFB1 concentrations were not significant, and no natural toxin concentration correlated significantly with these data. Only DON correlated negatively with the draught indices, but this year, only three genotypes showed higher DON values than the EU limit (1.75 mg/kg); the rest were close to zero or zero, so generally valid correlations were not found. No significant correlations were found with FAO numbers; this seemed to be indifferent. This result was important, as it allowed data to be merged for two years to be analyzed.

### 2.4. Summary of Traits

In the summary table (Table 14), the data means for isolates are presented for ear rots, toxigenic species, artificial (regular font), and natural infection and toxin contamination (*italic*) data. The colored highlights make it easier to interpret the data. Only P9415 showed a lower performance for all traits under the column mean (dark and light green, meaning that the stability was high (4739). Cadixxio DUO showed one case with a higher amount than the column means, but for aflatoxin, which had the lowest natural AFB1 contamination, its stability was good. Out of all the hybrids, these two can be grouped into the low–medium risk category, again with good stability (6416). As for the others, the highest susceptibility and best resistance occur in every hybrid for different traits. The variance also shows this. Fornad and Sy Zephyr could be discussed, and earlier data could be looked for, or another test could be included. Armagnac represents an especially alarming case; all toxin data are in the dangerous group, and for ear infections, only the value for Fg ear rot is lower than the mean for the column. Its instability is the worst (45,602).

The resistance (ear rot response) and resistance to toxins disagree in most cases (13A). We should mention that we have the mean responses to three isolates; therefore, the reliability of the data is significantly higher than that of data obtained from tests using only one inoculum. Of the 66 correlations, only 8 were significant. The AER correlated significantly with the FAO numbers; i.e., the later hybrids were more infected in this set. However, the AFB1 showed no correlation with FAO numbers (r = 0.025 and r = 0.259); i.e., there were other factors influencing AFB1 contamination. We never assumed that only one trait could explain the very complicated symptom/toxin relations, and now, this seems to be the correct version. In *F. graminearum*, we can see a significant correlation between disease symptoms and toxin contamination (r = 0.730). This is a tendency. For example, Cadixxio DUO is an FAO 470 hybrid with one of the lowest GER contaminations (1.88%), but its DON value is 5.68 mg/kg. The hybrid Armagnac, with nearly the same FAO number, showed a slightly higher GER with 9 mg/kg DON and 9 mg/kg FUM at a high FER severity. Our conclusion is that risk levels should be assessed for all traits separately, and, in the end, we should combine the results to make a suitable decision.

We also checked how the ear rot and toxin data relate to the rates of toxin contamination for one percentage of ear rot. For the 11 traits, 33 correlations were counted, of which only 1 was significant with r = 0.788 and *p* = 5%, between DON production at artificial inoculation and DON%/1% ear rot. There seem to be quite different regulations concerning DON production for 1% ear rot. Based on the correlation between traits shown in Table 12, it would have been a surprise to see a different result. However, this finding has significant implications for risk analysis in maize hybrids and also in inbred lines. This also has significance for the forecast of toxin contamination. When we know this phenomenon, we can work with it, even if the background is not clear.

### 2.5. Regression between Ear Rot Severity and Toxin Contamination

**Figure 1:**
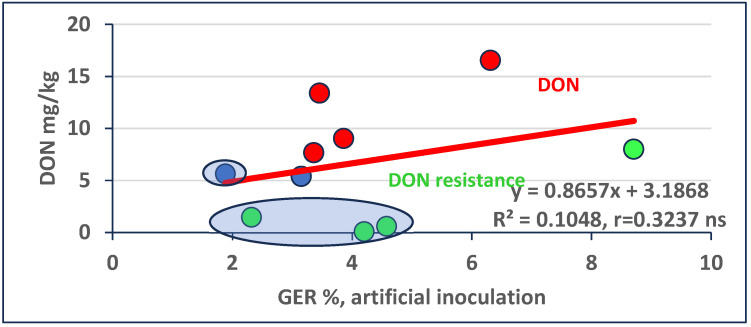
Regression between GER and DON contamination data in artificial inoculation across three isolates and two years (2021/2022). LSD 5% for DON = 0.24 mg/kg; for LSD 0.1% this value is 0.365. Red: overproduction, green: relative toxin resistance, blue: data on the regression line. Ellipses highlight: selected plus variants.

The regressions between ear rot values and mycotoxin contamination show very similar conclusions to those that we published in 2022 [30]. The difference is that the measuring of two subsamples for an isolate significantly decreases the “Within” value in ANOVA, and so the LSD values become smaller. This is important, as it allows us to see the toxin overproduction and lower production (relative DON resistance) data (Figure 1) more exactly. The data show a positive correlation between the two traits, but it is not significant. The reason is simple: at the same GER severity, considerably different levels of DON contamination are possible. We calculated the distance between the data point and its location on the regression line, making it possible to also calculate this value for all hybrids even if the GER and DON data are not vertically on the same line. Here, we identified four genotypes with higher and lower toxin overproduction (when LSD 5% was higher than the distance between the two data points) and, again, four genotypes with relative resistance (relative resistance to DON accumulation), e.g., below the regression line. Two remained very near to the regression line, just above the limit of significance between the data and the regression point. We highlighted four genotypes with lower GER and DON contamination from the present hybrid population; these seem to be useful variants. The highlighted hybrids are suggested for use in further testing.

**Figure 2:**
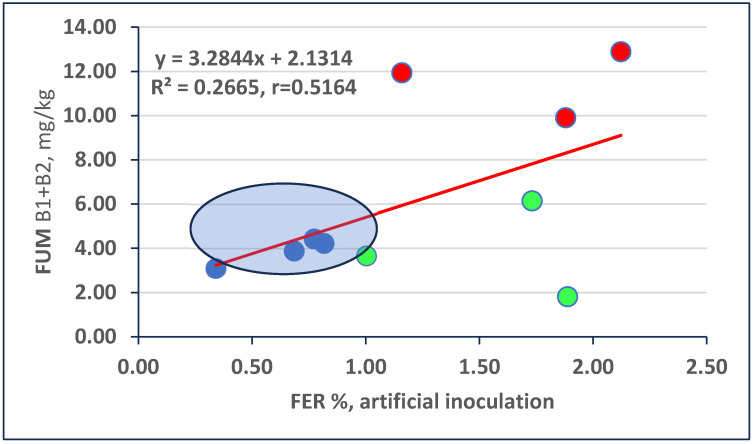
Regression between FER and FUM contamination data in artificial inoculation across three isolates and two years (2021/2022). LSD 5% for FUM = 0.13 mg/kg; for LSD 0.1% this value is 0.27. Red: overproduction, green: relative toxin resistance, blue: data on the regression line. Ellipsis highlight: selected plus variants.

The regression between FER and FUM contamination is closer to the significance limit that we found for DON, and the effects are the same: large and significant differences in FUM contamination and FER severity rate (Figure 2). The LSD 5% is for FUM 0.13, and the LSD 0.1% is 0.27. Four genotypes are within the distance of LSD 5% from the regression line. Three genotypes are significant FUM overproducers with significantly higher toxin contamination than would be predicted based on their ear severity. Three produced significantly less FUM than forecast by the regression line; this is the relative FUM resistance. The highlighted hybrids are suggested for further testing. 

**Figure 3:**
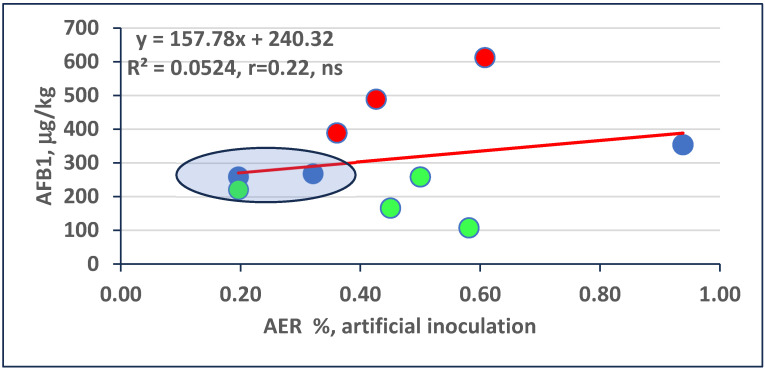
Regression between AER and AFB1 contamination data in artificial inoculation across three isolates and two years (2021/2022). LSD 5% for AFB1 = 28 μg/kg; for LSD 0.1% this value is 48.24 μg/kg. Red: overproduction, green: relative toxin resistance, blue: data on the regression line. Ellipsis highlight: selected plus variants.

For AFB1, we have a similar situation (Figure 3). We can see a group of low infected and low AFB1 contaminated groups very close to the regression line. There are three clean toxin overproducers and four hybrids with relative resistance to toxin contamination, all differing significantly between the data points and their corresponding points on the regression line. Three hybrids have regular responses. Their distance from the regression line is smaller than the LSD 5%.

### 2.6. Comparison of Ear Rot Severity and Toxin Contamination

The DON contamination was significantly less in the dry and hot 2022 than in 2021 (Table 15). The fumonisin was constant for artificial infection but doubled in the artificial infection regime. The aflatoxin increased fortyfold in 2022 compared to 2021 in artificial infection; in the natural regime, it rose ninefold, so this responded most sensitively to the change from normal to dry and hot weather. Significant correlations were identified only in five cases of the 66. This is not a decisive rate.

## 3. Discussion

### 3.1. Isolates and Resistance Responses

As discussed previously [13,28,30,37,38], the majority of studies have used only one inoculum for tests (single isolates or mixtures) with the assumption that the phenotyping would correspond to the genotypic data. We agree with the literature that no race-specific resistance has been identified in cereal Fusaria or Aspergilli until now. In our studies, we often found significant isolate/genotype interactions, but they were not stable, and this is the main argument against race-specific resistance to these pathogens. This was first mentioned by Tu [48]. However, the ranking of varieties normally changes when different isolates are used, and the differences are often significant; we can also see this in the contradictory data in this paper. At low aggressiveness, the genotype differences are not well expressed. Therefore, the value of the data from one isolate or inoculum is problematic. *The problem is the lack of stability across environments (years, isolates, ecology) cannot be counted and cannot be considered.* This is an important phenotyping problem. The data for the toxin data for the two years show this clearly. The question is, which are the correct data? Except for those for zero aggressiveness, all have meaning, and at higher aggressiveness, there is a higher probability of gaining useful data than in the opposite case. This means that all data are meaningful. Classic authors [49,50] are clear that each *Fusarium* species is a rather variable fungal population, where the individual isolates differ in many traits beyond aggressiveness. Therefore, one single isolate cannot represent the species, and the existing data [30] from two isolate tests and a test with 15 different isolates from 1982 [39] clearly show the highly variable responses of the hybrids to different isolates. The level of aggressiveness is important because we need to check not only the genotype ranking but also the amount of resistance. At higher aggressiveness, the genotype differences are more expressed.

Miedaner et al. [38] published an important paper in which 8–8 isolates were tested for *F. graminearum* and *F. verticillioides* for ear rots and toxin contamination in five inbred lines; two were characterized as resistant, and three as susceptible. These data prove to us that the mean response to more isolates is closer to the genetic difference than any one isolate on its own. For the same year, the correlations among responses to the isolates vary among the five genotypes. Figure S1 from Table 6 from [38] clearly shows the inbred differences in isolate responses of *F. graminearum*. Four inbreds are in the group that is resistant to isolate 8, and only D171 shows a significant difference from the others. UH303, which is classified as susceptible, is in the resistant group for isolate 8. For Isolate 2, the case is similar; for the others, the difference is much larger, and D171 is more susceptible but with a large difference. Regarding the correlations for inbreds across isolates, six were significant, and the other four were not. This picture is very similar to what we found in our tests, so their findings support our idea.

Looking at the correlations for the isolates counted for the five inbreds, the correlations are much closer; most of them were significant, indicating close responses; 15 were significant, and 13 were not. However, the limit for degree of freedom (df) was 3 and not six, as was seen for the 8 isolates (df = 6), and on this type of counting, the one isolate use could be supported.

For *F. verticillioides*, the inbred reactions in Germany did not correlate significantly to the eight isolates (Figure S2); the situation was worse when we counted for DON. On the other side, when the correlations were counted for the correlations of the inbred to the eight isolates, isolates (df = 3), the closeness of the correlations was significantly higher; 16 were significant, and 12 were not.

We compared the mean data for the two toxigenic species; the correlation was r = 0.55, with no significant correlation (Figure S3). The conclusion is that resistance to *F. graminearum* and *F. verticillioides* did not correlate significantly; at three inbreds, the data corresponded well, but the resistance of UH303 and D171 strongly differed. This agrees well with our conclusion that for individual genotypes, the resistance to the two toxigenic species can agree well, but this is not a general low.

The difference between the two correlation types is consequent. Actually, four inbreds belonged to the same resistance group, and only UH303 showed a real difference from them. The type of the two correlations (Data for inbreds and data for isolates) do not have the same meaning and are not interchangeable. In conclusion, that the means of the more isolates provide a more reliable genotype classification. QTL analysis, resistance classification, and all types of phenotyping will need more isolates. An additional advantage is that there is no environmental interaction. At the same time, more years and locations remain basic necessities. Gaikpa et al. [44] mentioned a number of problems of phenotyping; the isolated problem was not named. Of course, the extent of the work is tripled. With two evaluators, we could treat 80–100,000 ears in a season. So, this can be performed.

It seems to us that the analysis of the data supports our view that there are advantages to the use of more isolates in tests. We also found that, in agricultural production, it is necessary to perform a risk analysis to consider all possible aspects.

### 3.2. Change in Toxin Analysis

Earlier [28,29], all replicates of all maize resistance tests were subject to toxin analysis. Because of the costs, the replicates were pooled for an isolate, and a toxin analysis was made. However, in even more isolates and years, the varying data were treated by the ANOVA as replicates, and this often caused no significance between hybrids; even ten or morefold differences were recorded. A two-way ANOVA without replicates was able to reduce the Within value to some extent, but even so, the differences in toxin contamination were seldom significant. The solution was that two subsamples from the pooled sample were analyzed, and this way, the LSD value of the test decreased radically. The two subsamples reported a homogeneity for the sample that was normally less than ten percent between the two subsamples. This way, we could form a better opinion about the toxin over- or underproduction, and the finer differences could be investigated. This can lead to significant scientific and practical outcomes.

### 3.3. Stability in Resistance and Resistance to Toxin Accumulation

The idea of stability goes back to 2020 when eight hybrids were tested against three toxigenic species and three isolates for a toxigenic fungus. Toxin data were not made, but the symptom analysis showed that the three isolates gave a more reliable test and a wider possibility to stability analyses we started first in 2019. In the paper, several tables considered stability [30], but it is generally applied in this paper. When we realized the importance of this trait, it became a decisive role that was applied in all tables. This had several reasons.

We now have extensive data supporting the polygenic inheritance of ear rot resistances [27,45]. The yielding ability is also a polygenic trait; therefore, a variety of tests have been performed in many locations over several years for registration and other scientific purposes. This methodology works, and breeders can select higher-yielding varieties. In this work, three aspects are important. We need higher yields, yield stability under different conditions, and stability in resistance and toxin responses. Due to polygenic resistance, the toxigenic fungi require the same approach, and for this, a wider database is necessary in order to obtain reliable information on the stability of responses. One site, one isolate, and two years cannot yield a reliable dataset for the complex analysis of a trait.

As the responses of the hybrids to different isolates are not the same, a higher number of isolates should be used to correspond to some extent to the large variability of the fungal population tested [50,51]. The presented experimental material in this paper and the previous papers [13,28,29,30,37,38] support the usefulness of the idea. The stability of the hybrid responses to different pathogens shows this stability or instability well. Out of 10 hybrids, 1–3 was previously found to have good stability [28,29,30], and this is also true in this paper. As all epidemics are caused by natural epidemics, the increasing significance of natural infection cannot be underestimated [10].

The resistance and toxin contamination often differ; in about 50% of the genotypes, proportional symptoms and toxin contamination can be found, but in the rest, there are many possibilities. As no general resistance to different toxigenic species exists, we have to test resistance to them separately. In several hybrids, we found agreement in terms of resistance to *F. graminearum* and *F. verticillioides*, and 103 genes in the OGD family were identified [52]. These may play a role in resistance regulation, and there are QTLs that are common to *F. graminearum* and *F. verticillioides* resistance. In cases where resistance and susceptibility disagree, further research is necessary, and it is not known whether some of the genes are specific or common to the two diseases and their traits (symptoms and toxins). However, the existing experimental material allows the conclusion that different genetic regulations can be one valid hypothesis. This is supported by Alkohue and Miedaner [53], performing a meta-analysis of 15 papers and 224 QTLs in a dense genome-wide SNP for symptoms and the toxins *F. graminearum* and *F. verticillioides*. They identified 40 MQTL, and 19 of them were common to silk and kernel resistance and in FER and GER resistance. In total, 59 candidate genes out of 2272 were found to determine resistance to both *F. graminearum* and *F. verticillioides*. This explains the differences in resistance to the two pathogens and can explain them; they depend on the QTL in question. This topic is important, but nobody has applied this testing technology for commercial purposes. In this work, not only is the resistance ranking important, but the amount of resistance should also be stated. There is not a lot of research on this issue. We know that at low- or medium-aggressiveness, most genotypes will be more resistant, and maybe 10% maximum severity, most plants can be categorized as resistant. The same plant population against a highly aggressive infection would reach 70–80% infection severity, with several highly resistant plants. Our data show this clearly, and the two results cannot be true at the same time as genetically validated data and the Miedaner data [38] have stressed the complex consideration of more genetic agents by single and multi-trait genome-wide association mapping in wheat, but it is even more important for maize.

We have uncovered additional proof that toxin regulation and ear rot resistance regulation are often different; therefore, without toxin analysis, no food safety statement can be made. In 2022, we first reported in maize that the toxin production for a percentage of visual infection could differ very strongly, and, in several cases, high mycotoxin, mostly AFB1, was found in seemingly symptomless ears [30]. Munkvold [54,55] also reported this phenomenon. These also disturb the correlations between symptoms and toxin contamination. This is one cause of the mostly non-significant correlations between symptoms and toxin contamination, making it probable that a rather complicated mechanism is behind the phenomenon. The details of this are not known, and more research should be conducted to find better explanations than we currently have.

### 3.4. Epidemiological Patterns of Draught, Disease, and Toxin Relations

The weather data were rather different in the two years; 2021 was close to normal average temperature precipitation. The *F. graminearum* ear rot was the same in the two years, and the isolate means were very similar, without significant difference. As *F. graminearum* is highly aggressive, the coverage of the husk leaves could secure humidity in the first several weeks after artificial inoculation did not differ, but in other years also, a lower *F. graminearum* infection was recorded. The differences in natural infection are larger and more expressed [10]; in many hybrids, they were zero in 2022. The *F. verticillioides* infection was sixfold higher in the mean of years in dry years, and the same difference was seen also for *A. flavus* infection.

Toxins. DON responded very sensitively with an 80% reduction for artificial inoculation, and in 2022, the natural DON contamination was reduced to zero. The fumonisin contamination was stable in nature and doubled in natural infection. The most striking difference is seen in aflatoxin, where a 40-fold and 9-fold average increase of contamination happened at artificial and natural contamination with a simple hot summer. We found this also in 2019 and 2020 [30]. The differences were similar we found here. The other surprising matter is the severity of the contamination when 20 μg/kg is the limit, and we have maximum values in artificial inoculation close to 5000 μg/kg, and the natural maximum was higher than 2000 μg/kg, which shows the extent of the problem. In 2022, we found first that all hybrids were contaminated naturally higher than 20 μg/kg, even though we had an AFB1 difference between 28 and 585 μg/kg minimum and maximum in this test. In 2022, half of the hybrids did not contain any detectable aflatoxinB1. We know from [54,55] that the hot and dry weather increases aflatoxin contamination. It seems that years come with maximum and minimum aflatoxin contamination. So, a good previous crop and regular weather do not forecast anything for the next season. We agree that excellent water management and tillage help to keep AFB1 at a low level. However, there is seemingly enough *Aspergillus* population in soils that can be activated extremely rapidly. Under dry and hot conditions, natural DON actually disappears; under normal conditions, the limit values can easily surpass. Fumonisin is the most stable, but natural toxin contamination increases. In aflatoxin, the increase of toxin contamination is sixfold higher than we found for the symptoms. This response is much higher than we would conclude from the visual infection differences. This means that epidemiologically, they are very different, and beyond the resistance background, the epidemic characteristics should also be known. Such tests with the three fungal species can contribute to the epidemiological behavior of the different hybrids, as they really behave differently.

This supports the view [54,55] that an extra aflatoxin increase can be the rule for many hybrids. As we excluded insect-damaged ears from disease and toxin evaluation, our data clearly proves and demonstrates the disease and its environmental effects without this background noise. In no other paper have I found such a methodical approach. Maybe they did, but it was never mentioned. For these articles, we cannot know the role of insect damage in the presented toxin results, so the year and environmental data may also not be reliable. There is no general epidemic pattern for maize for the different ear rots. Every hybrid has its own pattern; this is mostly resistance-dependent. The year data show this clearly. This is the reason that without more resistant hybrids, no solution is possible; even the best combination of agronomic practices and resistance promises a 100% reduction in toxin contamination. However, it may allow economic maize production.

### 3.5. Food Safety Risk Analysis

In breeding, we can use many different ideas, approaches, methodologies, traditional and molecular techniques, or their combinations, and the result will be a genotype that should be tested for its food safety risk. As tests were performed in two locations and over two years for each toxigenic spp., we have 9 datasets from artificial injection and 3 from natural infection for one year; for the two sites and two years, we have 36 artificial infection datasets and 12 natural infection datasets (48 altogether), and this means 16 datasets for statistical analyses. When a third year is necessary, the number of data will increase to 72 and 24 replicate means for statistical tests. For all toxigenic species, this provides a database that allows for a much better food safety evaluation than those previously available. It will also provide a dataset for scientific research. This number of data enables a rather good evaluation of toxin contamination risks in maize hybrids.

What is the basic information for the risk analysis?

1. We need to know the amount of resistance in any given genotype to each of the toxigenic species that we have to deal with under artificial inoculation trials with three different isolates tested previously for aggressiveness and toxin production.
2. We need to know the genotypes’ toxin responses to all three toxigenic fungi in artificial inoculation trials with three different isolates tested previously for aggressiveness and toxin production. The minimum comprises DON, FUM B1 + B2, and AFB1 for all treatments; this way, the toxin interactions can be determined.
3. We need to know the level of infection severity for *Fusarium* spp. and *Aspergillus* spp. separately.
4. We need to know the amount of natural toxin contamination assessed via the multitoxin method for DON, FUM B1 + B2, AFB1 + AFG1, ZEN, T-2, and HT-2.
5. We mentioned the significance of natural infections; a multitoxin screening will follow two other registration tests, and two replicates from each location will be tested.
6. Each year, seeds will be collected from toxigenic species for identification and toxin tests to build a microbe gene bank to store and test isolates.
7. We have to select control varieties in the different ripening groups for both disease and toxin resistance to help better classify the hybrids under sometimes strongly variable environmental conditions. We can cite here the resistant control Korimbos, which showed mostly low severity numbers but far less optimistic results for toxins [10,30].

What do we have to consider?

As the trade and human consumption of maize and its use in animal feeding are all based on toxin limits, the most important trait is toxin contamination. For natural infection, the toxin limits are decisive. Hybrids with toxin contamination above limit values represent a higher risk. The question is, how high are these numbers?

A high infection severity is enough to withdraw a hybrid from further tests, even if the toxin contamination is lower than expected (relative resistance to toxin accumulation). The reason for this is that when an isolate with much better toxin production can cause severe problems. The same is true for more resistant but highly toxin-contaminated hybrids because these can be identified only through toxin analysis.

It is good when the artificial and natural infection data agree; when they disagree, we posit that the natural toxin data should have higher significance. In the opposite case, when the artificial contamination is high, but the natural contamination is low, we may decide to perform further tests. In ANOVA, significant interactions might also be significant. A larger dataset will help in finding the best solution, and we are sure that in several years, we will achieve a much higher exactness in our data than we currently have.

Risk analysis is also important when we want to decide whether a hybrid is suitable for baby food or young animal feed, where limits are about 20% of the limits for adults or developed animals. In the dairy industry, aflatoxin is critical; therefore, in this case, somewhat higher DON or fumonisin may be tolerated better. As different animals often have differing toxin risks, this information can be utilized by the feed industry.

The Hungarian Agricultural Ministry has suggested the introduction of this registration methodology in Hungary; the date of introduction depends on financial resources.

### 3.6. Is Resistance Alone a Comprehensive Solution?

According to all the relevant literature, there is no immunity against toxigenic fungi in maize. In spite of this, highly significant resistance differences exist among hybrids. The efficacy of resistance can be increased using different practices, which can further reduce the toxin contamination achieved by the more resistant hybrids. This can further reduce the losses due to toxin contamination. At present, no effective fungicide technology exists; this should be developed. Cropping system management is also useful; however, crop rotation, tillage, planting dates, irrigation, and fertilization have shown limited or moderate efficacy [56]. Therefore, the results for susceptible hybrids are doubtful. Insect, weed, and harvest date management can also help; in this, Bt genes play a role [55]. Over 10 years, we conducted fungicide tests in maize to reduce toxin contamination, but the work on susceptible hybrids did not lead to significant toxin reduction in artificially inoculated tests. A 10 mg/kg DON reduction compared to the untreated control at 70 mg/kg has no practical value when the limit for pigs is 0.9 mg/kg. As the works were funded by Plant Protection Entertainments, we could not publish the results, but lessons for the future have been made.

In wheat, the highly susceptible varieties could not be protected effectively against FHB and toxins [57,58], but a medium-resistant cultivar combined with fungicide treatment gave very promising results. Willyerd et al. [59] stressed the importance of the combination of resistance and fungicide application. We concluded that the susceptible and highly susceptible cultivars must be withdrawn from commercial production. As in maize, there is no acceptable fungicide protection methodology; the susceptible and highly susceptible hybrids must also be eliminated from commercial production. This is the reason for the high significance of the official hybrid resistance tests. This will make it possible for farmers to select only from low and low-to-medium-risk hybrids for commercial production.

Breeding programs will be updated, and more and more resistant hybrids can be registered. The testing of hybrids in breeding firms applying this methodology, or a simplified one, can identify the new combinations providing high yield and high resistance. As the inbreds behind the hybrids are known, the inbreds can be identified as having a role in the more resistant hybrids. This will help in planning new combinations to improve yield and resistance to toxigenic fungi. We have to know that a higher resistance in inbreds does not necessarily mean good combining ability for the trait [60].

## 4. Materials and Methods

### 4.1. Plant Materials and Experimental Design

Over two years, 10 hybrids were tested from an international collection; they are listed in Table 1. All hybrids were dent types, so I gave only the FAO numbers that are more or less stable for a hybrid, at least in a region. In the variety description booklets as cited, no toxin information is presented, and no comparable kernel infection is given. The grain, silo, or mixed-use is known but can change for the same hybrid in Italy, Hungary, and France, for example, and alternative utilization is also the case. In a risk analysis, we need to know first what the resistance level and its stability are, irrespective of the genetic background difference and breeding methods of the given hybrids and another trait, as all form the end result. This is, therefore, because the grower is interested in one thing: to produce well-selling, high-yielding, and toxic contamination below the limit. It is the problem of the breeding and how it solves this problem. This is similar to yielding ability; we can define hybrids with multiyear and multilocation tests, which have higher yield potential. This is also the case here. The control varieties were the same as those that we used earlier [30], with Korimbos as the resistant control and DKC4541 as the susceptible control. The experiments were conducted as part of the Trials System of the Hungarian Maize Club Association (a public organization, www.magyarkukoricaklub.hu [61], which has the objective of improving the information available to farmers regarding the usefulness of the hybrids that they grow.

The experimental conditions, fertilization, and plant protection were as described earlier [30]. Before 2017, we worked in four m-long rows with 15–18 plants per row. However, rain or irrigation did not always come in time; we planted 8 m long rows with 30 plants to secure the necessary 15–18 plants for the experiments for harvest. So, the experimental area for the hybrids doubled, but even under less suitable conditions, the experiment could be evaluated normally, and the ear number was comparable to, or sometimes larger than, that which could be obtained in the shorter plots. Irrigation was a possibility after sowing 30–40 mm of water to secure uniform germination; later, the water supply could not be optimized.

### 4.2. Isolates and Inoculation

The isolates were the same as those that we listed [30] for *F. graminearum* No. Fg6 was added to the isolates No. Fg 3, Fg4, the isolates of *F. verticillioides* (Fv1, Fv5) were supported by isolate Fv8, and Af3 was added to the two *A. flavus* isolates (Af1, Af2). Their identification (traditional and molecular), aggressiveness test, and the inoculation method (toothpick) have been reported in detail [30]. This is the first paper performed with three isolates per toxigenic species. Inoculation was performed using the toothpick method [38,62] as this caused about 3.5-fold higher ear infection and toxin contamination than the silk channel method did.

### 4.3. Evaluation of Symptoms and Risks

For all toxigenic species, a percentage scale was used scale was used [28,38]. There are many other options [37], but the reason for this change was to ensure the correct comparison of the toxin contamination with the visually evaluated symptom severity. Using different scales for different disease agents would make it impossible to compare them. For evaluation of the ear infection severity most sensitive scale should be used. Normally, there are 7–800 grains in a regular ear, with variations. When only 1 grain is infected in an ear, this represents 0.15%, 7 grains represent 1%, 70 grains correspond to 10%, and about 100 grains correspond to 15%, and then the steps follow by 10% until reaching 100. For artificial inoculation, only grains that are infected around the fungus-inoculated toothpicks should be considered. Other *Fusarium* or *Aspergillus* (green) infections that were seemingly due to independent natural infections were rated separately. Insect-damaged ears were not considered, as we wanted to determine the resistance/susceptibility relations without the influence of insects. Several ears were also harvested from the inoculated row that did not show marks from toothpicks; this happens when there is a mistake with the inoculation. These ears were also not considered. However, ears with toothpick marks without infection were rated with zero. For toxin analysis, only insect-free ears were considered. In the literature, no detailed evaluation of this has been presented until now.

In risk evaluation, the column means are decisive. Low risk corresponds to 0–50% of the mean and is shown in dark green; a value between 51 and 100% of the mean, shown in light green, highlights low-to-medium risk; yellow designates medium-to-high risk (100–150%); and all remaining genotypes belong to the high-risk group, shown in orange. Visually, this makes it easier to consider the risk matrix of the hybrids. For natural toxin contamination, we also applied the EU toxin limit values [21,22,23,24,25].

### 4.4. Sample Preparation for Toxin Analyses

The work stages were reported earlier [30]. Sampling is a very important aspect; how can we produce the high level of representation needed for the required reliability? Traditional subsampling, with 100 subsamples for every 500 tons of grain, could not be used, so a 10 kg sample will represent the 500 tons. This is a 1:50,000 rate that is impossible for such an experimental situation. From each row, we selected five ears for toxin analysis, that is, 30–40% of the successfully harvested and identified inoculation points and infection around it. The yield of the five years is between 0.8 and 1.2 kg, depending on the hybrid, and the sample has a rate of about 1:3 compared to 1:50,000. In one kg of grain, 3–4000 kernels can be found depending on 1000 grain mass. At a low infection level, grain sampling is problematic because of the chance is high to find manyfold differences in 100 g subsamples. Especially *A. flavus*, when only 5–6 aflatoxin-contaminated grains occur in a kg, accurate sampling is not possible. For this reason, the whole kg was roughly milled to 1–2 mm particles, meaning that a much higher homogeneity was achieved than would be achieved by traditional sampling of whole grains, with three 100 g samples from 1 kg for each replicate. This milled grain was thoroughly mixed, and 100 g was separated for analysis. Then, the 3 × 100 g was pooled and mixed carefully, and 100 g was taken from it in Szeged and sent to city Nagyigmand, the Bonafarm-Babolna Feed Ltd., Laboratory Branch for toxin analyses. The sampling rate for the three replicates is 1:30 for the whole outgoing material, and the rough milling improves the sampling precision significantly, so the preciosity is much better than for the praxis prescribed at 1:50,000. The laboratory is accredited for all mycotoxin measurements [30].

### 4.5. Toxin Analysis

For the analyses, the methodology was the same as described earlier [30]. There is one important difference: from each pooled sample, two samples were analyzed, obtaining two independent measurements for the given sample [10], where the data for 2021 were published by this methodology. This way, we could check the homogeneity of the pooled samples, and the Within value for the ANOVA became significantly smaller; the two datasets normally did not have a difference larger than 10 percent.

### 4.6. Statistical Methods

The basic methods were the same as presented earlier [30]. The difference is that in the toxins, we had two subsamples; the toxin AVOVA became four-way, three-way, and in several cases, two-way tests were used. The functions counting the main and effect and their interaction were described by Svab [63] and Weber [64]. In this way, a deeper insight into the problem of toxin interactions, its overproduction, and ear-rot x toxin analyses becomes possible.

## 5. Conclusions

The resistance levels of maize to toxigenic species mostly differ; therefore, the testing of resistance to the three major fungal species requires separate resistance and toxin tests. This was also verified in this test. As all pathogenic populations consist of highly variable individual lines with differing characteristics, the polygenic nature of resistance and, in many cases, the weak relation between symptoms and toxin contamination mean that toxin control is needed in every case. Therefore, as in yield tests, a rather wide dataset is needed here in order to correctly estimate both the resistance differences and the amount of resistance. The increase in the number of independent isolates to three increased the data by 50%. This allowed us to introduce stability analyses for the traits. This is decisive; this trait was never investigated from this point of view. The data will allow us to make more precise disease and toxin predictions, as the resistance level can be considered in forecast models. This is not currently possible, as there are no data. A better methodology is important for genetic research; phenotyping could be much more correct than it currently is. This would make validation easier. Of course, this would also cost more money. This methodology can be applied to check fungicide control bots for disease and toxin reduction. In mass breeding work, a simplified methodology is possible, but at the stage of preparing hybrids for variety registration, a complete methodology is suggested. As disease severity and toxin regulation mostly differ, both should be followed. As between hybrids, highly manyfold resistance differences exist. This allows the selection of the best and excludes the susceptible and highly susceptible hybrids from commercial production. We think that, by this, the presently 300–350 million tons of global loss can be decreased by at least 100 million tons, and with the breeding of even more resistant varieties, this can increase further. The higher resistance supported by other practices is the key element in increasing food safety and yield [57,58], as was found in wheat.

## Figures and Tables

**Table 1:** Ear rot data (%) of artificial inoculation tests on maize hybrids. 2021–2022.

Hybrid	*F. graminearum*			*F. verticillioides*			*A. flavus*			Check	Mean	Variance
	Fg3	Fg4	Fg6	Fv1	Fv5	Fv8	Asp1	Asp2	Asp3	Fus. spp.		
Cadixxio Duo	1.62	2.46	1.55	0.76	0.81	0.48	0.56	0.31	0.09	0.54	0.86	0.46
P9415	3.88	1.28	1.79	1.05	0.76	0.51	0.20	0.24	0.15	0.66	0.99	1.05
SY Zephir	2.26	4.87	2.95	0.29	0.18	0.56	0.10	0.15	0.34	0.33	1.06	2.28
Fornad	4.51	4.69	4.51	1.23	0.80	0.98	0.45	0.31	0.32	0.49	1.61	3.27
DKC 5542	1.20	4.10	4.16	3.04	1.28	1.31	0.40	0.82	0.52	0.83	1.61	1.84
Korimbos	5.06	3.91	1.39	1.47	1.13	0.87	0.18	0.68	0.64	2.52	1.91	2.12
P9978	3.98	5.32	3.28	2.32	1.58	1.29	0.74	0.37	0.17	1.39	1.94	2.37
Armagnac	2.87	3.17	5.53	1.68	3.40	1.29	0.40	0.77	0.65	1.43	2.01	2.18
P 0725	8.87	6.90	3.17	0.44	0.98	1.03	0.60	0.68	1.54	0.49	2.14	7.95
DKC 4541	6.67	8.58	10.86	1.53	2.66	1.48	0.45	0.60	0.29	0.91	2.99	13.06
Mean	4.09	4.53	3.92	1.38	1.36	0.98	0.41	0.49	0.47	0.96	1.71	
LSD 5%											0.73	
**Correlations**	**Fg3**	**Fg4**	**Fg6**	**Fv1**	**Fv5**	**Fv8**	**Asp1**	**Asp2**	**Asp3**			
Fg4	0.653 *											
Fg6	**0.298**	0.695 *										
Fv1	−0.322	−0.004	0.233									
Fv5	0.118	0.246	0.675	**0.426**								
Fv8	0.273	0.614	0.723 *	0.679 *	0.744 *							
Asp1	0.247	0.345	0.185	0.267	0.272	0.464						
Asp2	0.216	0.2636	0.324	0.536	0.618	0.699 *	0.1815					
Asp3	0.631	0.368	−0.032	−0.204	0.071	0.230	0.136	**0.575**				
Check	0.069	−0.074	−0.090	0.403	0.389	0.292	−0.114	**0.478**	**0.055**			
**ANOVA**												
**Source**		**MS**		**df**		**MQ**			**F**		**LSD 5%**	
Hybrid A		266.5		9		29.62			5.98 ***		0.73	
Year B		139.8		1		139.83			28.24 ***			
Isolate C		7.7		2		3.84			0.78			
Toxigenic. spp. D		1522.1		3		507.38			102.50 ***		0.46	
A x B		307.0		9		34.11			6.89 ***			
A x C		59.3		18		3.29			0.67			
A x D		528.1		27		19.56			3.95 ***			
B x C		1.2		2		0.58			0.12			
B x D		171.0		3		57.01			11.51 ***			
C x D		10.4		6		1.74			0.35			
A x B x C		68.1		18		3.78			0.76			
A x B x D		454.6		27		16.84			3.40 ***			
A x C x D		286.8		54		5.31			1.07			
B x C x D		30.3		6		5.06			1.02			
A x B x C x D		160.1		54		2.97			0.60			
Within		2377.4		480		4.95						
Total		6390.6		719								

**bold**: correlations between isolates responses within species. Fg = *F. graminearum*. Fv = *F. verticillioides* and Af = *Aspergillus flavus*. * *p* = 0.05. *** *p* = 0.001.

**Table 2:** The *F. graminearum* (Fg) data ear rot data (ear coverage %) of artificial inoculation in maize from the two years 2021–2022.

Hybrid		2021			2022			
	Fg3	Fg4	Fg6	Fg3	Fg4	Fg6	Mean	Variance
**Cadixxio Duo**	0.64	0.93	1.31	2.60	4.00	1.79	1.88	1.3
**P9415**	5.52	1.30	1.86	2.23	1.26	1.72	2.32	2.2
DKC 5542	0.99	2.62	1.80	1.40	5.58	6.52	3.15	4.5
SY Zephir	3.91	8.79	5.64	0.62	0.94	0.26	3.36	9.7
Korimbos	7.18	5.61	0.45	2.94	2.22	2.33	3.46	5.1
Armagnac	0.90	1.13	1.28	4.83	5.22	9.79	3.86	10.1
P9978	2.07	2.91	2.94	5.90	7.74	3.62	4.20	3.9
Fornad	4.19	6.76	4.63	4.83	2.62	4.40	4.57	1.5
P 0725	14.30	10.41	4.42	3.43	3.39	1.92	6.31	20.1
DKC 4541	6.85	10.19	13.16	6.50	6.98	8.56	8.71	5.6
Mean	4.66	5.06	3.75	3.53	4.00	4.09	4.18	
LSD 5%							2.53	
**ANOVA**								
**Source**	**SS**	**df**	**MS**	**F**	***p*-Value**	**F Crit.**	**LSD 5%**	
Hybrid A	653.56	9	72.62	4.83	1.6207 × 10^−5^	1.96	2.53	
Isolate/year B	49.75	5	9.95	0.66	0.65	2.29	ns	
AxB	1100.30	45	24.45	1.63	0.02	1.47	6.20	
Within	1802.34	120	15.02					
Total	3605.96	179						
**Correlations**								
**Isolates**	**Fg3**	**Fg4**	**Fg6**	**Fg3**	**Fg4**	**Fg6**	**Mean**	
Fg4	0.7269 *							
Fg6	0.3131	0.7256 *						
Fg3	0.0687	0.1422	0.4506					
Fg4	−0.2667	−0.1142	0.2891	0.6807 *				
Fg6	−0.2905	−0.1131	0.2905	0.5823	0.6630 *			
Mean	0.5530	0.7601 *	0.8503 **	0.6546 *	0.4404	0.4412	1	

** *p* = 0.01. * *p* = 0.05. Fg = *F. graminearum*. Bold: low infection, high stability.

**Table 3:** The *F. verticillioides* ear rot data (ear coverage %) of artificial inoculation in maize from the two years 2021–2022.

Hybrid		2021			2022			
	Fv1	Fv5	Fv8	Fv1	Fv5	Fv8	Mean	Variance
**Sy Zephir**	0.22	0.10	0.13	0.35	0.25	0.98	0.34	0.11
**Cadixxio Duo**	0.17	0.10	0.21	1.35	1.52	0.76	0.68	0.40
**P9415**	0.20	0.23	0.14	1.89	1.28	0.88	0.77	0.50
**P0725**	0.31	1.29	0.73	0.57	0.67	1.33	0.82	0.17
**Fornad**	0.33	0.46	0.52	2.12	1.15	1.44	1.00	0.49
Korimbos	0.20	0.23	0.08	2.75	2.04	1.66	1.16	1.31
P9978	0.14	0.27	0.35	4.51	2.89	2.23	1.73	3.19
DKC 5542	0.20	0.27	0.38	5.89	2.29	2.25	1.88	4.82
DKC4541	0.44	0.22	0.60	2.62	5.09	2.35	1.89	3.51
Armagnac	0.21	0.60	0.95	3.14	6.19	1.63	2.12	5.04
Mean	0.24	0.38	0.41	2.52	2.34	1.55	1.24	
LSD 5%							0.86	
**ANOVA**								
**Source**	**SS**	**df**	**MS**	**F**	***p*-Value**	**F Crit.**	**LSD 5%**	
Hybrid A	61.76	9	6.86	3.98	0.00018	1.96	0.86	
Isolate/year B	161.18	5	32.24	18.71	1.03 × 10^−13^	2.29	0.66	
AxB	131.67	45	2.93	1.70	0.01222	1.47		
Within	206.76	120	1.72					
Total	561.36	179						
**Correlations**	**Fv1**	**Fv5**	**Fv8**	**Fv1**	**Fv5**	**Fv8**		
Fv5	0.3031							
Fv8	0.4444	0.6767 *						
Fv1	−0.2764	−0.2386	0.0878					
Fv5	0.1887	−0.0379	0.6127	0.4557				
Fv8	0.2436	−0.0052	0.3352	0.7713 *	0.5808			
Mean	0.0974	0.0252	0.5646	0.7961 **	0.8690 ***	0.8463 **		

*** *p* = 0.001. ** *p* = 0.01. * *p* = 0.05, Fv = *F. verticillioides*. Bold: low infection, high stability.

**Table 4:** The *A. flavus* ear rot data (ear coverage %) of artificial inoculation in maize from the two years 2021–2022.

Hybrid		2021			2022		Mean	Variance
	Asp1	Asp2	Asp3	Asp1	Asp2	Asp3		
**Sy Zephyr**	0.04	0.06	0.04	0.17	0.24	0.64	0.20	0.05
**P9415**	0.03	0.04	0.07	0.37	0.44	0.22	0.20	0.03
**Cadixxio Duo**	0.07	0.03	0.04	1.05	0.59	0.14	0.32	0.17
**Fornad**	0.20	0.14	0.12	0.70	0.48	0.52	0.36	0.06
P9978	0.18	0.02	0.10	1.30	0.73	0.24	0.43	0.24
DKC4541	0.19	0.18	0.12	0.72	1.03	0.47	0.45	0.13
Korimbos	0.11	1.17	0.06	0.25	0.19	1.22	0.50	0.29
DKC 5542	0.15	0.02	0.08	0.65	1.62	0.95	0.58	0.39
Armagnac	0.20	0.08	0.06	0.61	1.46	1.24	0.61	0.37
P0725	0.21	0.10	0.08	1.00	1.25	3.00	0.94	1.27
Mean	0.14	0.18	0.08	0.68	0.80	0.86	0.46	0.30
**ANOVA**								
**Source**	**SS**	**df**	**MS**	**F**	**p-Value**	**F crit.**	**LSD 5%**	
Hybrid A	7.87	9	0.87	2.03	0.04	1.96	0.43	
Isolate/year B	19.67	5	3.93	9.15	2.1224 × 10^−7^	2.29	0.33	
AxB	25.61	45	0.57	1.32	0.12	1.47	ns	
Within	51.58	120	0.43					
Total	104.73	179						
**Correlations**	**Asp1**	**Asp2**	**Asp3**	**Asp1**	**Asp2**	**Asp3**		
Asp2	−0.0606							
Asp3	0.6797 *	−0.0994						
Asp1	0.5128	−0.4306	0.3749					
Asp2	0.6334	−0.4117	0.2074	0.3758				
Asp3	0.4480	0.1775	−0.1081	0.0494	0.4192			
Mean	0.7221 *	0.0973	0.1432	0.3699	0.6854 *	0.8853 ***		

*** *p* = 0.01. * *p* = 0.05, Af = *A. flavus* Bold: low infection, high stability.

**Table 5:** Summary of the artificial inoculation data of maize ear rots (ear rot severity as %) across years and isolates. 2021–2022.

Hybrid		Toxigenic spp.			Mean	Variance
	Fg	Fv	Af	Check		
**Cadixxio Duo**	**1.88**	0.68	0.32	0.54	0.86	0.49
**P9415**	2.32	0.77	0.20	0.66	0.99	0.85
**SY Zephir**	3.36	0.34	0.20	0.33	1.06	2.37
Fornad	4.57	1.00	0.36	0.49	1.61	3.99
DKC 5542	3.15	1.88	0.58	0.83	1.61	1.37
Korimbos	3.46	1.16	0.50	2.52	1.91	1.77
P9978	4.20	1.73	0.43	1.39	1.94	2.57
Armagnac	3.86	2.12	0.61	1.43	2.01	1.91
P 0725	6.31	0.82	0.94	0.49	2.14	7.77
DKC 4541	8.71	1.89	0.45	0.91	2.99	14.88
Mean	4.18	1.24	0.46	0.96	1.71	
LSD 5%	2.53	0.86	0.43	1.06	0.73	
**Correlations**	**Fg**	**Fv**	**Af**	**K**	**Mean**	
Fv	0.3537					
Af	0.4430	0.3604				
Check	−0.0408	0.4455	0.1765			
Mean	0.895 ***	0.663 *	0.5652	0.3494		

*** *p* = 0.001. * *p* = 0.05. Fg: *F. graminearum*. Fv: *F. verticillioides*. Af: *A. flavus*. Dark green: low. light green: low to medium. yellow: medium to high. and orange: high toxin risk. Bold: Genotypes with good resistance and high stability (low variance).

**Table 6:** Natural *Fusarium* ear rot infection (%) data on the control and artificially inoculated ears. 2021–2022.

Hybrid	Toxigenic spp./Isolates									Check			Mean
	Fg3	Fg4	Fg6	Fv1	Fv5	Fv8	Asp1	Asp2	Asp3	1	2	3	
**P 0725**	0.12	0.08	0.39	0.05	0.07	0.05	0.04	0.05	0.02	0.11	0.09	0.19	0.10
**Cadixxio Duo**	0.09	0.20	0.11	0.07	0.13	0.11	0.17	0.07	0.08	0.11	0.11	0.09	0.11
**SY Zephir**	0.18	0.11	0.10	0.19	0.20	0.08	0.08	0.13	0.39	0.15	0.13	0.18	0.16
**Korimbos**	0.06	0.04	0.07	0.06	0.12	0.03	0.09	0.05	1.85	0.03	0.02	0.63	0.25
P9415	0.07	0.16	0.36	0.25	0.30	0.18	0.61	0.49	0.22	0.30	0.31	0.25	0.29
DKC 5542	0.06	0.62	0.07	0.57	0.43	0.15	0.43	0.37	0.18	0.34	0.39	0.07	0.31
Armagnac	0.19	0.31	0.25	0.27	0.19	0.18	0.43	0.85	0.27	0.28	0.34	0.18	0.31
DKC 4541	0.07	0.52	0.29	0.33	0.38	0.22	0.66	0.20	0.20	0.34	0.38	0.26	0.32
Fornad	0.31	0.17	0.30	0.30	0.62	0.10	0.14	0.15	0.62	0.36	0.42	0.45	0.33
P9978	0.62	1.49	0.17	0.18	0.32	0.92	0.95	0.24	0.23	0.57	0.69	0.45	0.57
Mean	0.18	0.37	0.21	0.23	0.28	0.20	0.36	0.26	0.40	0.26	0.29	0.27	0.28
LSD 5% Hybr.													0.19
LSD 5% column													0.15
**Correlations**	**Fg3**	**Fg4**	**Fg6**	**Fv1**	**Fv5**	**Fv8**	**Asp1**	**Asp2**	**Asp3**	**1**	**2**	**3**	
Fg4	0.74 *												
Fg6	0.01	−0.15											
Fv1	−0.10	0.28	−0.07										
Fv5	0.28	0.29	0.12	0.709 *									
Fv8	0.85 **	0.94 ***	−0.04	0.04	0.18								
Asp1	0.48	0.82 **	0.13	0.37	0.31	0.81 **							
Asp2	0.02	0.15	0.20	0.45	0.10	0.12	0.44						
Asp3	−0.11	−0.28	−0.40	−0.28	−0.10	−0.23	−0.32	−0.26					
Check1	0.69 *	0.83 **	0.18	0.54	0.68 *	0.79 **	0.83 **	0.36	−0.39				
Check2	0.71 *	0.84 **	0.15	0.53	0.66 *	0.80 **	0.83 **	0.39	−0.36	0.99 ***			
Check3	0.39	0.12	−0.05	−0.34	0.15	0.25	0.05	−0.28	0.79 **	0.08	0.11		
Mean	0.72 *	0.837 **	−0.01	0.36	0.53	0.83 **	0.82 **	0.34	0.04	0.88 ***	0.90 ***	0.45	

*** *p* = 0.001. ** *p* = 0.01. * *p* = 0.05. Fg: *F. graminearum*. Fv: *F. verticillioides*. Af: *A. flavus.* Highlighting: dark green: low. light green: low to medium. yellow: medium to high. and orange: high toxin risk.

**Table 7:** Natural *Fusarium* ear rot infection (%) data on the control and artificially inoculated ears. 2021/2022.

Hybrid	Fg	Fv	Af	K	Mean	Variance
P 0725	0.20	0.06	0.03	0.13	0.10	0.01
Cardixxio Duo	0.13	0.11	0.11	0.10	0.11	0.00
SY Zephir	0.13	0.16	0.20	0.15	0.16	0.00
Korimbos	0.06	0.07	0.66	0.23	0.25	0.08
P9415	0.20	0.24	0.44	0.28	0.29	0.01
DKC 5542	0.25	0.38	0.32	0.27	0.31	0.00
Armagnac	0.25	0.21	0.52	0.27	0.31	0.02
DKC 4541	0.29	0.31	0.35	0.32	0.32	0.00
Fornad	0.26	0.34	0.30	0.41	0.33	0.00
P9978	0.76	0.47	0.48	0.57	0.57	0.02
Mean	0.25	0.24	0.34	0.27	0.28	
**Correlations**	**Fg**	**Fv**	**Af**	**K**		
Fv	0.7900 **					
Af	0.1868	0.2687				
K	0.8473 **	0.8678 **	0.5003			
Mean	0.8484 **	0.8627 **	0.6236	0.9658 ***		

*** *p* = 0.001. ** *p* = 0.01, Fg: *F. graminearum*. Fv: *F. verticillioides*. Af: *A. flavus*.

**Table 8:** Natural *Aspergillus* ear rot infection (%) data on the control and artificially inoculated ears. 2021/2022.

	Fg	Fv	Af	K	Mean	Variance
SY Zephir	0.08	0.01	0.18	0.20	0.12	0.01
Korimbos	0.08	0.17	0.28	0.00	0.13	0.01
Fornad	0.08	0.19	0.29	0.00	0.14	0.02
P9415	0.18	0.05	0.23	0.11	0.14	0.01
Cadixxio Duo	0.23	0.20	0.39	0.08	0.23	0.02
DKC 4541	0.37	0.02	0.40	0.13	0.23	0.03
Armagnac	0.14	0.25	0.63	0.11	0.28	0.06
P 0725	0.09	0.02	1.04	0.08	0.31	0.24
P9978	0.27	0.16	0.58	0.34	0.34	0.03
DKC 5542	0.36	0.44	0.57	0.29	0.41	0.02
Mean	0.19	0.15	0.46	0.13	0.23	
LSD 5% Hybrid	0.26	0.27	0.46	0.25	0.16	
LSD 5% tox. spp.					0.10	

Fg: *F. graminearum*. Fv: *F. verticillioides*. Af: *A. flavus*.

**Table 9:** The mycotoxin contamination in artificial inoculation resistance tests to toxigenic fungi for the three isolates per toxigenic spp. 2021–2022.

Hybrid	DON mg/kg				Fumonisin B1 + B2 mg/kg				AFB1 mg/kg				Mean	Variance
	Fg3	Fg4	Fg6	Ch.	Fv1	Fv5	Fv8	Ch.	Af1	Af2	Af3	Ch.		
**Fornad**	**1.26**	0.30	0.33	0.42	2.00	5.29	3.73	1.64	0.934	0.056	0.176	0.145	1.36	2.67
**P9415**	0.88	0.09	3.55	0.00	6.63	1.54	5.16	1.38	0.154	0.462	0.049	0.087	1.66	5.01
P9978	0.05	0.14	0.20	0.06	3.39	11.19	3.85	3.10	0.191	1.257	0.020	0.199	1.97	10.57
DKC 4541	4.63	7.84	11.60	0.00	1.09	3.45	0.89	0.35	0.386	0.106	0.006	0.087	2.54	14.03
**Cadixxio Duo**	9.30	3.09	4.65	0.07	2.82	4.54	4.33	3.21	0.448	0.353	0.005	0.028	2.74	7.79
SY Zephir	13.33	7.10	2.61	0.00	3.12	3.11	3.07	2.28	0.233	0.527	0.020	0.240	2.97	14.86
DKC 5542	10.63	1.86	3.73	2.38	9.68	11.24	8.79	7.48	0.147	0.126	0.050	0.029	4.68	20.60
P 0725	21.77	18.83	9.05	0.00	3.18	6.15	3.39	2.87	0.082	0.899	0.080	0.585	5.57	55.12
Armagnac	24.95	0.62	1.59	1.75	7.34	13.17	18.16	7.26	0.985	0.840	0.011	0.434	6.42	67.69
Korimbos	16.12	7.12	16.98	0.07	1.85	12.62	21.35	1.37	0.298	0.139	0.338	0.052	6.52	64.38
Mean	10.29	4.70	5.43	0.47	4.11	7.23	7.27	3.09	0.386	0.476	0.075	0.188	3.64	26.27
LSD 5%													0.086	
LSD 5% Col													0.094	
**ANOVA**														
**Source**		**MS**			**df**		**MQ**			**F**			**F _AxBxCxD_**	
Hybrid A		1693.0			9		188.11			4089.5			2.61 *	
Year B		685.0			1		685.00			14,891.2			9.51 **	
Isolate C		959.0			3		319.67			6949.4			4.43 **	
Toxins. D		2715.1			2		1357.57			29,512.3			18.84 ***	
A x B		970.6			9		107.84			2344.3			1.50	
A x C		1406.1			18		78.12			1698.2			1.08	
A x D		2278.7			18		126.59			2752.0			1.76	
B x C		884.9			3		294.98			6412.6			4.10 *	
B x D		2174.2			2		1087.08			23,632.3			15.09 ***	
C x D		1540.2			6		256.71			5580.6			3.56 **	
A x B x C		1605.5			27		59.46			1292.7			0.83	
A x B x D		1608.8			27		59.58			1295.3			0.83	
A x C x D		2660.5			54		49.27			1071.1			0.68	
B x C x D		1676.7			6		279.46			6075.1			3.88 **	
A x B x C x D		3889.3			54		72.02			1565.8				
Within		10.9			240		0.046							
Total		26,758.7			479									
**Correlations**														
	**Fg3**	**Fg4**	**Fg6**	**Check**	**Fv1**	**Fv5**	**Fv8**	**Check**	**Af1**	**Af2**	**Af3**	**Check**		
Fg4	0.508													
Fg6	0.292	0.584												
Check	0.328	−0.351	−0.295											
Fv1	0.196	−0.381	−0.411	0.818 **										
Fv5	0.431	−0.167	0.088	0.578	0.329									
Fv8	0.567	−0.148	0.350	0.407	0.271	0.755 *								
Check	0.479	−0.260	−0.406	0.906 ***	0.813 **	0.622	0.384							
Af1	0.133	−0.42	−0.314	0.277	−0.055	0.188	0.287	0.198						
Af2	0.198	0.101	−0.371	−0.114	0.108	0.249	−0.063	0.232	−0.16					
Af3	0.100	0.131	0.568	−0.149	−0.313	0.322	0.564	−0.307	0.062	−0.400				
Check	0.637 *	0.548	−0.121	0.007	0.029	0.131	0.017	0.226	0.104	0.655 *	−0.18			
Mean	0.902 ***	0.412	0.490	0.418	0.254	0.675 *	0.775 **	0.484	0.027	0.091	0.347	0.419		

*** *p* = 0.001. ** *p* = 0.01. * *p* = 0.05. Fg: *F. graminearum*. Fv: *F. verticillioides*. Af: *A. flavus*. Highlighting: dark green: low. light green: low to medium. yellow: medium to high. and orange: high toxin risk.

**Table 10:** The DON contamination in resistance tests to *F. graminearum* and artificially contaminated checks. 2021–2022.

Hybrid	DON mg/kg 2021				DON mg/kg 2022				Mean	Variance
	Fg3	Fg4	Fg6	Check	Fg3	Fg4	Fg6	Check		
**P9978**	**0.10**	0.00	0.00	0.12	0.00	0.29	0.41	0.00	0.11	0.02
**Fornad**	2.53	0.61	0.67	0.83	0.00	0.00	0.00	0.00	0.58	0.74
**P9415**	1.77	0.19	7.10	0.00	0.00	0.00	0.00	0.00	1.13	6.19
Cadixxio Duo	18.60	6.10	9.30	0.14	0.00	0.08	0.00	0.00	4.28	46.18
DKC 5542	21.26	3.73	0.82	4.77	0.00	0.00	6.64	0.00	4.65	51.47
SY Zephir	26.66	14.20	5.22	0.00	0.00	0.00	0.00	0.00	5.76	96.32
DKC 4541	9.26	15.49	21.69	0.00	0.00	0.19	1.51	0.00	6.02	72.64
Armagnac	49.89	1.24	1.81	3.50	0.00	0.00	1.36	0.00	7.22	298.63
Korimbos	32.23	14.23	5.57	0.14	0.00	0.00	28.38	0.00	10.07	180.96
P 0725	43.54	37.51	16.32	0.00	0.00	0.16	1.78	0.00	12.41	334.16
Mean	20.58	9.33	6.85	0.95	0.00	0.07	4.01	0.00	5.22	108.73
LSD 5%									0.20	
**Correlations**	**Fg3**	**Fg4**	**Fg6**	**Check**		**FG4**	**Fg6**			
Fg4	0.5119									
Fg6	0.1008	0.6931 *								
Check	0.3288	−0.3480	−0.4673							
Fg4	−0.2785	0.2392	0.3754	−0.3901						
Fg6	0.2800	0.1581	−0.0819	0.0173		−0.2632				
Mean	0.8489 **	0.8241 **	0.4849	0.0057		−0.0709	0.4656			

** *p* = 0.01. * *p* = 0.05. Data for Fg3 and Fg Check were all below the detection limit designated by 0. Therefore, the correlations could be counted. Fg: *F. graminearum.* Highlighting: dark green: low. light green: low to medium. yellow: medium to high. and orange: high toxin risk. Bold: high resistance and high stability (low variance).

**Table 11:** The FUM B1 + B2 contamination in artificially inoculated resistance tests to *F. verticillioides* and naturally contaminated checks. 2021–2022.

Hybrid	FUM B1 + B2, mg/kg, 2021				FUM B1 + B2, mg/kg, 2022					Variance
	Fv1	Fv5	Fv8	Check	Fv1	Fv5	Fv8	Check	Mean	
**DKC 4541**	1.07	3.95	0.50	0.61	1.12	2.95	1.29	0.09	1.45	1.8
**SY Zephyr**	5.56	3.33	0.62	0.71	0.69	2.88	5.53	3.85	2.89	4.3
Fornad	2.68	9.29	5.89	2.34	1.32	1.28	1.58	0.94	3.16	8.6
P9415	1.58	2.68	1.61	2.12	11.67	0.40	8.71	0.63	3.67	17.3
**Cadixxio Duo**	0.69	7.08	4.77	0.59	4.94	1.99	3.88	5.83	3.72	5.8
P 0725	3.80	10.84	2.56	0.40	2.57	1.46	4.22	5.33	3.90	10.3
P9978	1.49	5.04	1.41	1.76	5.30	17.34	6.29	4.43	5.38	27.0
Korimbos	1.87	15.65	35.33	0.28	1.82	9.59	7.36	2.46	9.29	137.3
DKC 5542	4.73	1.65	14.49	0.83	14.64	20.83	3.09	14.14	9.30	57.4
Armagnac	10.55	20.79	10.39	0.65	4.14	5.56	25.94	13.87	11.48	72.8
Mean	3.40	8.03	7.75	1.03	4.82	6.43	6.79	5.16	5.42	
LSD 5%									0.11	
**Correlations**	**Fv1**	**Fv5**	**Fv8**	**Check**	**Fv1**	**Fv5**	**Fv8**	**Check**		
Fv5	0.5304									
Fv8	0.0616	0.5025								
Check	−0.2432	−0.3268	−0.3724							
Fv1	0.0028	−0.4105	0.0234	0.2510						
Fv5	0.0638	−0.1603	0.3573	−0.0537	0.4967					
Fv8	0.778 **	0.7027 *	0.1645	−0.1324	0.0364	−0.0196				
Check	0.7218 *	0.2857	0.1896	−0.3490	0.4533	0.5254	0.5314			
Mean	0.6096	0.6045	0.6885 *	−0.2933	0.3249	0.5587	0.6753 *	0.7597 **		

** *p* = 0.01. * *p* = 0.05. Highlighting: dark green: low. light green: low to medium. yellow: medium to high. and orange: high toxin risk. Bold: high resistance and good stability (low variance).

**Table 12:** The AFB1 contamination in artificially inoculated resistance tests to *A. flavus* and naturally contaminated checks. 2021–2022.

Hybrid	AFB1 2021. μg/kg				AFB1 2022. μg/kg				Mean	Variance
	Af1	Af2	Af3	Check	Af1	Af2	Af3	Check		
**DKC 5542**	**58.8**	13.8	0.0	2.5	88.3	112.3	49.5	26.3	43.9	1679
DKC 4541	234.3	13.3	0.8	0.0	152.0	93.0	4.8	86.8	73.1	7373
**P9415**	0.0	5.0	0.0	0.0	154.3	457.0	48.5	87.0	94.0	24,564
Korimbos	79.3	9.8	0.0	2.5	218.3	129.3	338.3	49.0	103.3	14,600
**Cardixxio Duo**	3.3	13.5	0.0	0.0	444.8	339.8	5.0	27.5	104.2	32,473
SY Zephir	2.5	74.0	0.0	0.0	230.8	452.5	19.5	240.0	127.4	27,372
Fornad	54.5	50.8	0.0	0.0	879.8	5.3	176.3	145.0	163.9	88,088
P 0725	5.0	10.8	0.0	0.0	77.0	888.3	80.3	584.8	205.8	114,967
P9978	23.0	5.3	0.0	0.0	167.5	1251.5	19.5	199.3	208.3	184,000
Armagnac	10.0	7.8	0.0	0.0	975.3	832.3	10.8	433.8	283.7	169,742
Mean	47.1	20.4	0.1	0.5	338.8	456.1	75.2	187.9	140.8	63,141.8
LSD 5%									15.4	
Column									13.8	
**Correlations**	**Af1**	**Af2**	**Af3**	**Check**	**Af1**	**Af2**	**Af3 1**	**Check**		
Af2 B1	−0.112									
Af3 B1	0.9189 ***	−0.109								
Check	0.162	−0.197	−0.167							
Af1 B1	−0.177	0.208	−0.200	−0.299						
Af2 B1	−0.496	−0.300	−0.309	−0.428	−0.060					
Af3 B1	0.089	0.021	−0.234	0.590	0.042	−0.394				
Check	−0.351	0.008	−0.190	−0.424	0.181	0.6458 *	−0.185			
Mean	−0.423	−0.064	−0.323	−0.481	0.565	0.7287 *	−0.119	0.7937 *		

*** *p* = 0.001. * *p* = 0.05. Highlighting: dark green: low. light green: low to medium. yellow: medium to high. and orange: high toxin risk. Bold: high resistance and good stability (low variance).

**Table 13:** Ear rot resistance tests in maize, rate of toxin contamination for a percentage of visual ear rot severity, 2021/2022.

Hybrid	DON mg/kg/1/% ER	FUM mg/kg/1/% ER	AFB1 mg/kg/1% ER
Cadixxio Duo	3.02	5.69	3225
P9415	0.65	5.75	1994
SY Zephyr	2.29	9.12	1299
Fornad	0.14	3.66	**ZD**
DKC 5542	1.72	5.27	376
Korimbos	3.88	10.30	**ZD**
P9978	0.03	3.55	1451
Armagnac	2.35	6.07	5723
P 0725	2.62	5.20	4245
DKC 4541	0.92	0.96	1294
Mean	1.76	5.56	2451
Max/Min	129.33	10.73	15.23

ZD = zero divider.

**Table 14:** Summary table of the resistance tests of maize hybrids to toxigenic fungi across years and isolates, 2021–2022.

Hybrid	Ear Rot %, Toxigenic spp.					DON mg/kg		Fum B1 + B2 mg/kg		AFB1 mg/kg		FAO Nr.	Variance
	Fg	Fv	Af	F nat.	Af nat.	Fg Art.	Fg nat.	Fv art	Fv nat	Af art.	Af nat.		
**Cadixxio Duo**	**1.88**	0.68	0.32	0.54	0.083	5.68	0.07	3.89	3.21	269	28	470	6416
**P9415**	2.32	0.77	0.20	0.66	0.111	1.51	0.00	4.44	1.38	222	87	350	4739
SY Zephyr	3.36	0.34	0.20	0.33	0.200	7.68	0.00	3.10	2.28	260	240	390	10,084
Fornad	4.57	1.00	0.36	0.49	0.005	0.63	0.42	3.67	1.64	389	145	421	14,509
DKC5542	3.15	1.88	0.58	0.83	0.286	5.41	2.38	9.90	7.48	108	29	535	1002
Korimbos	3.46	1.16	0.50	2.52	0.005	13.40	0.07	11.94	1.37	258	52	575	5892
P9978	4.20	1.73	0.43	1.39	0.337	0.13	0.06	6.14	3.10	489	199	390	23,354
Armagnac	3.86	2.12	0.61	1.43	0.107	9.05	1.75	12.89	7.26	612	434	491	45,602
P 0725	6.31	0.82	0.94	0.49	0.083	16.55	0.00	4.24	2.87	354	585	561	38,174
DKC4541	8.71	1.89	0.45	0.91	0.128	8.02	0.00	1.81	0.35	166	87	370	2835
Mean	4.18	1.24	0.46	0.96	0.134	6.81	0.47	6.20	3.09	313	188		15,261
LSD 5%	2.53	0.86	0.43	1.06	0.247	0.24	0.08	0.13	0.38	28	30		
**Correlations**	**Fg**	**Fv**	**Af**	**F nat.**	**Af nat.**	**Fg art.**	**Fg nat.**	**Fv art.**	**Fv nat.**	**Af art.**	**Af nat.**		
Fv	0.354												
Af	0.443	0.360											
F nat.	−0.041	0.446	0.176										
Af nat.	−0.066	0.347	−0.061	−0.114									
Fg art.	0.324	−0.077	0.679 *	0.264	−0.367								
Fg nat.	−0.197	0.618	0.298	0.083	0.304	−0.048							
Fv art.	−0.338	0.516	0.338	0.740*	0.009	0.255	0.647 *						
Fv nat.	−0.331	0.502	0.373	0.043	0.406	0.030	0.906 ***	0.649 *					
Af art.	−0.027	0.247	0.229	0.211	−0.045	−0.046	0.060	0.320	0.277				
Af nat.	0.317	−0.002	0.653 *	−0.148	−0.060	0.507	0.007	0.066	0.227	0.616			
FAO Nr.	−0.093	0.106	0.730 *	0.411	−0.253	0.705 *	0.381	0.627	0.437	0.026	0.259		

*** *p* = 0.001. * *p* = 0.05. Highlighting: dark green: low. light green: low to medium, yellow: medium to high, and orange: high toxin risk, Fg *= F. graminearum*, Fv = *F. verticillioides*, Af = *A. flavus.* Bold: high resistance and good stability (low variance).

**Table 15:** Mycotoxin contamination averages in maize resistance tests, 2021, 2022.

Hybrid	DON Art. mg/kg		DON Natural		Fum Art mg/kg		Fum Nat mg/kg		Afla Art mg/kg		Afla Natural	
	2021	2022	2021	2022	2021	2022	2021	2022	2021	2022	2021	2022
P9415	1.51	0.00	0.00	0.00	1.96	6.92	4.29	0.63	0.00	439	0.	174
DKC 4541	7.74	0.57	0.00	0.00	1.84	1.78	4.45	0.09	25.	166	77	173.
P9978	0.02	0.23	0.12	0.00	2.65	9.64	6.45	4.43	15.	959	45.	398
SY Zephir	7.68	0.00	0.00	0.00	3.17	3.03	3.95	3.85	1.	468	5	480
Fornad	0.63	0.00	0.83	0.00	5.95	1.39	2.34	0.94	7.	707	21	290
Cadixxio Duo	5.67	0.03	0.14	0.00	4.18	3.60	0.59	5.83	1.8	526	5.	55
Armagnac	8.82	0.45	3.50	0.00	13.91	11.88	0.65	13.87	6.	1212	19	867
DKC 5542	4.30	2.21	4.77	0.00	6.95	12.85	0.83	14.14	35.	166.	105	52.
Korimbos	8.67	9.46	0.14	0.00	17.62	6.26	0.28	2.46	44.	457.	134	98.
P0725	16.23	0.64	0.00	0.00	5.73	2.75	0.40	5.33	3.5	697.	10	1169
Mean	6.13	1.36	0.95	0.00	6.39	6.01	2.42	5.1	14.	580.	42	375
**Toxin**	**Year**	**DON Artificial**		**DON Natural**		**Fum Artificial**		**Fum Natural**		**Afla Artificial**		**Afla Nat**
**Correlations**		**2021**	**2022**	**2021**	**2022**	**2021**	**2022**	**2021**	**2022**	**2021**	**2022**	**2021**
		**mg/kg**	**mg/kg**	**mg/kg**	**mg/kg**	**mg/kg**	**mg/kg**	**mg/kg**	**mg/kg**	**mg/kg**	**mg/kg**	**mg/kg**
DON Art	2022	0.213										
DON Nat	2021	−0.057	0.013									
	2022	nd	nd	nd								
Fum Art	2021	0.325	0.765*	0.358	nd							
	2022	−0.219	0.148	0.757	nd	0.355						
Fum Nat	2021	−0.559	−0.411	−0.407	nd	−0.668	−0.086					
	2022	0.180	−0.034	0.885	nd	0.375	0.769 *	−0.486				
Af Art	2021	0.022	0.801 **	0.286	nd	0.507	0.303	−0.189	0.110			
	2022	0.010	−0.216	0.073	nd	0.292	0.269	−0.013	0.280	−0.428		
Af Nat	2021	0.022	0.801 **	0.286	nd	0.507	0.303	−0.189	0.110	1.000	−0.428	
	2022	0.639 *	−0.278	0.005	nd	0.113	−0.026	−0.175	0.247	−0.448	0.623	−0.448

** *p* = 1%, * = *p* = 5%. Fum = Fumonisin B1 + B2, Afla = aflatoxin B1, Art = artificial, Nat = Natural, not detected, zero divider.

## Data Availability

The data that support the findings of this study are available in the cited literature and the corresponding author upon reasonable request.

## Supplementary Materials

The following supporting information can be downloaded at: https://www.mdpi.com/article/10.3390/toxins16090390/s1, Figure S1: *F. graminearum* resistance of five maize inbred lines to eight *F. graminearum* isolates, source of the original data Miedaner et al. [38]; Figure S2: *F. verticillioides* resistance of five maize inbred lines to eight *F. verticillioides* isolates, source of original data: Miedaner et al. [38]; Figure S3: Comparison of the GER and FER resistance of the five inbreds based on data from the Miedaner paper [38].

## Abbreviations

AER	*Aspergillus* ear rot
AFB1	aflatoxin B1
ANOVA	analysis of variance
df	degree of freedom
DON	deoxynivalenol
FAO	Food and Agricultural Organization of United Nations
FB_1_	fumonisin B_1_
FER	*Fusarium* ear rot
FHB	*Fusarium* head blight
FUM	fumonisin(s)
GER	*Gibberella* ear rot
KWS	German Plant Breeding firm
MQTL	meta-QTL
QTL	quantitative trait locus
SNP	single nucleotide polymorphism
ZEN	zearalenone
