# Supplementary material for: Stability of Resistance of Maize to Ear Rots (Fusarium graminearum, F. verticillioides and Aspergillus flavus) and Their Resistance to Toxin Contamination and Conclusions for Variety Registration
Source: Toxins (Basel). 2024 Sep 10;16(9):390. doi: 10.3390/toxins16090390 (PMC11435759; doi:10.3390/toxins16090390)
Supplement: Supplementary file 1 [file toxins-16-00390-s001.zip › toxins-3150573-supplementary.pdf]

toxins

Stability of Resistance of Maize to Ear Rots (*Fusarium graminearum, F. verticillioides* and *Aspergillus flavus*) and Their Resistance to Toxin Contamination and Conclusions for Variety Registration

Akos Mesterhazy [1,*], Balazs Szabo [1], Denes Szieberth [2], Szabolcs Tóth [3], Zoltan Nagy [1], Tamas Meszlenyi [1], Beata Herczig [4], Attila Berenyi [1] and Beata Tóth [1]

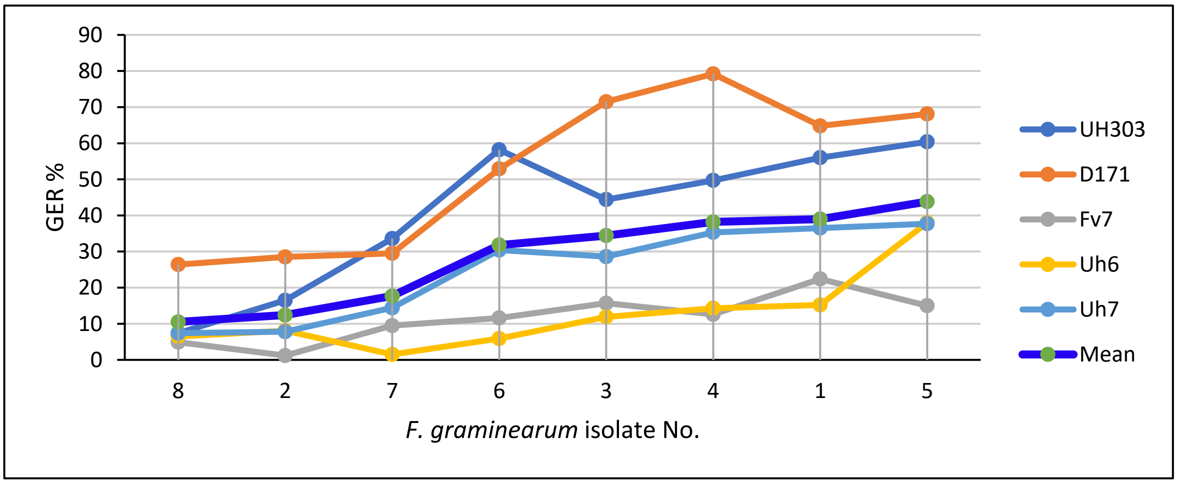

Inbreds	UH303	D171	Fv7	Uh6
UH303				
D171	0.8083*			
Fv7	0.8140*	0.7680*		
Uh6	0.5293	0.5788	0.4337	
Uh7	0.9514*	0.9273*	0.8624**	0.6273
** P = 0.01, * = 0.05				

Figure S1. *F. graminearum* resistance of five maize inbred lines to eight *F. graminearum* isolates, source of the original data Miedaner et al. [38].

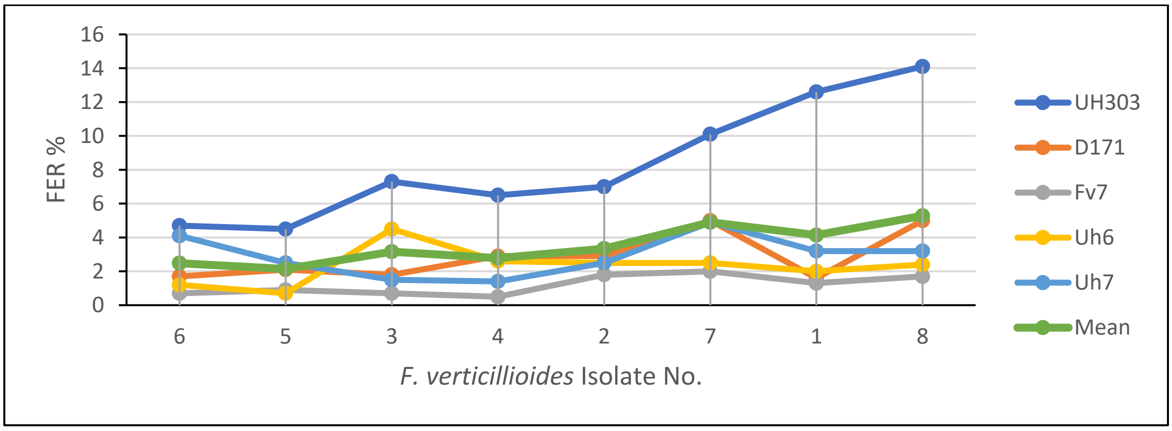

Inbreds	UH303	D171	Fv7	Uh6	Uh7
UH303					
D171	0.5387				
Fv7	0.6033	0.7023			
Uh6	0.2384	0.1084	0.0044		
Uh7	0.2815	0.4110	0.5766	-0.4161	
	0.9256*	0.7628	0.7744	0.2860	0.4649

P = 0.05.

Figure S2. *F. verticillioides* resistance of five maize inbred lines to eight *F. verticillioides* isolates, source of original data: Miedaner et al. [38].

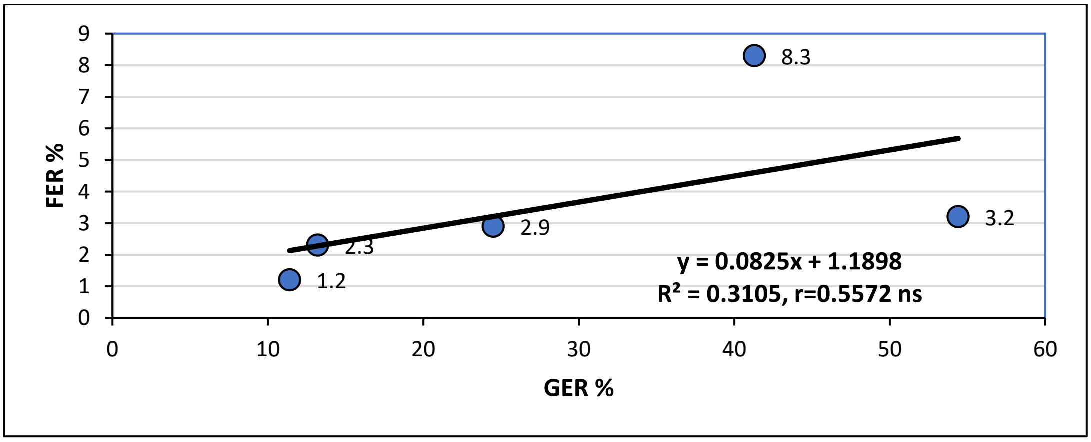

Figure S3. Comparison of the GER and FER resistance of the five inbreds based on data from the Miedaner paper [38].
